# CIRP promotes the progression of non-small cell lung cancer through activation of Wnt/β-catenin signaling via CTNNB1

**DOI:** 10.1186/s13046-021-02080-9

**Published:** 2021-08-31

**Authors:** Yi Liao, Jianguo Feng, Weichao Sun, Chao Wu, Jingyao Li, Tao Jing, Yuteng Liang, Yonghui Qian, Wenlan Liu, Haidong Wang

**Affiliations:** 1 The Central Laboratory, Shenzhen Second People’s Hospital/First Affiliated Hospital of Shenzhen University Health Science Center, Shenzhen, Guangdong 518035 P. R. China; 2 Department of Thoracic Surgery, Southwest Hospital, Army Medical University, Chongqing, 400038 P. R. China; 3 Department of Anesthesiology, The Affiliated Hospital of Southwest Medical University, Province, Luzhou, 646099 Sichuan China; 4 Department of Cardiology, Southwest Hospital, Army Medical University, Chongqing, 400038 P. R. China; 5 Department of Thoracic Surgery, Shenzhen Second People’s Hospital/First Affiliated Hospital of Shenzhen University Health Science Center, Shenzhen, Guangdong 518035 P. R. China

**Keywords:** Non-small cell lung cancer, CIRP, CTNNB1, Wnt/β-catenin

## Abstract

**Background:**

Cold-inducible RNA binding protein (CIRP) is a newly discovered proto-oncogene. In this study, we investigated the role of CIRP in the progression of non-small cell lung cancer (NSCLC) using patient tissue samples, cultured cell lines and animal lung cancer models.

**Methods:**

Tissue arrays, IHC and HE staining, immunoblotting, and qRT-PCR were used to detect the indicated gene expression; plasmid and siRNA transfections as well as viral infection were used to manipulate gene expression; cell proliferation assay, cell cycle analysis, cell migration and invasion analysis, soft agar colony formation assay, tail intravenous injection and subcutaneous inoculation of animal models were performed to study the role of CIRP in NSCLC cells; Gene expression microarray was used to select the underlying pathways; and RNA immunoprecipitation assay, biotin pull-down assay, immunopurification assay, mRNA decay analyses and luciferase reporter assay were performed to elucidate the mechanisms. The log-rank (Mantel-Cox) test, independent sample T-test, nonparametric Mann-Whitney test, Spearman rank test and two-tailed independent sample T-test were used accordingly in our study.

**Results:**

Our data showed that CIRP was highly expressed in NSCLC tissue, and its level was negatively correlated with the prognosis of NSCLC patients. By manipulating CIRP expression in A549, H460, H1299, and H1650 cell lines, we demonstrated that CIRP overexpression promoted the transition of G1/G0 phase to S phase and the formation of an enhanced malignant phenotype of NSCLC, reflected by increased proliferation, enhanced invasion/metastasis and greater tumorigenic capabilities both in vitro and in vivo. Transcriptome sequencing further demonstrated that CIRP acted on the cell cycle, DNA replication and Wnt signaling pathway to exert its pro-oncogenic action. Mechanistically, CIRP directly bound to the 3′- and 5′-UTRs of CTNNB1 mRNA, leading to enhanced stability and translation of CTNNB1 mRNA and promoting IRES-mediated protein synthesis, respectively. Eventually, the increased CTNNB1 protein levels mediated excessive activation of the Wnt/β-catenin signaling pathway and its downstream targets C-myc, COX-2, CCND1, MMP7, VEGFA and CD44.

**Conclusion:**

Our results support CIRP as a candidate oncogene in NSCLC and a potential target for NSCLC therapy.

**Supplementary Information:**

The online version contains supplementary material available at 10.1186/s13046-021-02080-9.

## Background

Non-small cell lung cancer (NSCLC) is one of the most malignant tumors, with an increasing incidence worldwide [1]. Although the diagnostic and treatment algorithms for NSCLC are constantly improving, it is difficult to obtain satisfactory curative effects, and the long-term survival rate is still low [2]. A relatively poor understanding of the molecular mechanisms underlying the pathogenesis of NSCLC is one of the reasons. Although, many studies have focused on the identification of NSCLC associated genes [3–5], the molecular pathogenesis of NSCLC is far from clear. Therefore, searching for new genes that are involved in the development of NSCLC is critical for improving the molecular typing and completing the regulation network of NSCLC, and may provide novel targets for precision therapy [6].

Cold-inducible RNA binding protein (CIRP) is constitutively expressed in the nucleus at low levels in various tissues, and its expression can be induced by mild hypothermia, UV irradiation, hypoxia, osmotic pressure, and ischemic disorders [7]. CIRP contains an RNA recognition motif (RRM) domain with RNA binding function and a glycine-rich C-terminal domain involved in cell localization [8]. Study have shown that sepsis and hemorrhagic shock can also induce CIRP to be released into extracellular (eCIRP) through the lysosomal secretion, and act as a damaged-associated molecular pattern (DAMP )[9]. Hitherto, several signaling pathways of inflammation induced by eCIRP have been uncovered. Wang et al. found that TREM-1 and TLR4-MD2 complexes act as eCIRP receptors to activate the inflammatory cascade and amplify the inflammation of macrophages [9, 10]; while the combination of eCIRP and IL-6R induces activation of the STAT3 pathway to make macrophages tend to the M2 phenotype, which may prevent the amplification of inflammation providing a negative feedback loop [11]. Whether eCIRP has biological effects other than inflammation regulation has not been studied yet.

Intracellular, it is believed that CIRP is a stress-induced protein and participates in multiple cellular signaling pathways by post-transcriptional regulation of the translation of specific mRNAs. Studies have shown that tumor necrosis factor-alpha (TNF-α), cycloheximide, or hypothermia treatment could upregulate the expression of CIRP and thus increase the expression of phosphorylated extracellular signal-regulated kinase 1/2 (ERK1/2), leading to the inhibition of the apoptosis signal acquisition factor caspase-3 and the bypassing of replicative senescence [12–16]. In addition, CIRP could post-transcriptionally regulate the expression of tumoral biomarkers RPA2 and TRX [17, 18], mediating the expression of poor prognosis-related genes such as hypoxia inducible transcription factor-1 (HIF-1) and vascular endothelial growth factor (VEGF) [19–21]. Moreover, studies have also reported that CIRP upregulation increases the expression of cell cycle-related proteins, such as cyclin D1 and c-Myc [22, 23]. In another study, CIRP was shown to negatively regulate p53 levels, thereby downregulating proapoptotic genes and upregulating antiapoptotic genes [24]. These findings all support CIRP as a new proto-oncogene to promote the progression of various cancers through multiple cellular signaling pathways, such as brain cancer, breast cancer, oral cancer, colon cancer, prostate cancer, and liver cancer [25]. However, the mechanisms underlying the role(s) of CIRP in the regulation of NSCLC progression remain largely unknown.

In this study, we investigated CIRP expression and its functions in NSCLC. Our data showed that CIRP was overexpressed in NSCLC cell lines and human NSCLC tissue, and CIRP overexpression was correlated with a poor prognosis in NSCLC patients. Mechanistically, CIRP directly bound to the 3′- and 5′-UTRs of CTNNB1 mRNA to enhance its stability and translation, and the resultant accumulation of CTNNB1 protein induced excessive activation of Wnt/β-catenin signaling and promoted the progression of NSCLC.

## Materials and methods

### Tissue arrays

Eighty-six tumor tissues and their paired noncancerous tissues were randomly collected from NSCLC patients who had undergone surgical resection in the Department of Thoracic Surgery, Southwest Hospital, Army Medical University (Chongqing, China). No antitumor treatment was performed before the surgery. All samples were frozen in liquid nitrogen within 10 min following surgical resection and stored at − 80 °C until analyses. Tissue array blocks containing NSCLC tissues and adjacent noncancerous tissues were established using a tissue microarrayer (Leica, Germany). Procedures for the collection of human samples and their usage for tissue arrays were approved by the Ethical Committee of the Army Medical University (Chongqing, China). Informed consent forms were obtained from the patients before participating in this study.

### Immunohistochemistry (IHC) and hematoxylin-eosin (HE) staining

The streptavidin-biotin peroxidase complex method was used for immunohistochemical staining of tissue array slides and formalin-fixed, paraffin embedded tissue sections. Antigen retrieval was performed by heating the dewaxed and dehydrated sections in Dako antigen retrieval solution containing 10 mM EDTA (pH 8.0) with a pressure cooker. Goat anti-human CIRP antibody (ab106230, Abcam, USA; 1: 250 dilution) was used to detect CIRP expression. Rabbit anti-human CTNNB1 antibody (19807S, Cell Signaling Technology, USA; 1: 200 dilution) was used to detect CTNNB1 expression. The expression of CIRP and CTNNB1 was evaluated using graded semiquantitative scoring system. The intensity of staining was classified as none (0), weak (1), strong (2) or very strong (3), and the staining patterns were classified into negative (0: ≤ 10%), sporadic (1: 11 to 25%), focal (2, 26 to 50%) or diffuse (3, ≥ 51%). An overall score was calculated by multiplying intensity and positivity scores as follows: 0 (negative), 1 and 2 (weak staining), 3 and 4 (moderate staining), and 6 and 9 (strong staining). To analyze clinical significance and prognosis, malignant samples with strong CIRP/CTNNB1 staining were classified as high-expression, whereas low-expression was indicated by moderate staining, weak staining and negative staining. Paraffin-embedded tumor sections were used for examination of HE staining following standard protocols. Photographs were taken using a light microscope.

### Cell culture

Human lung cancer cell lines A549, NCI-H1299, NCI-H1650, NCI-H460 and NCI-H446 were purchased from SUYANBIOTECH (Guangzhou, China). A549 cells with stable luciferase expression (A549-Luc^+^) were purchased from AIPONUO (Guangzhou, China). HEK293FT and HaCaT cells were obtained from Dr. Tang’s Lab (Chongqing, China). All cells were identified by short tandem repeat (STR) profiling and cultured according to the manufacturer’s specifications for less than 3 months. For the stable selection of H1299/Le-CIRP and H1650/Le-CIRP cells, the culture medium was supplemented with 2.0 μg/mL puromycin (#P8833, Sigma-Aldrich, MO, USA).

### Animal experiments

Female BALB/c nude mice (4-weeks old) were purchased from the Beijing Huafukang Bioscience Co., Ltd. (Beijing, China), and maintained in the Experimental Animal Center of Army Medical University (Chongqing, China). All procedures for animal experiments were approved by the Committee on the Use and Care on Animals (Army Medical University, Chongqing, China) and performed in accordance with institutional guidelines. After adaptive feeding for 2 weeks, pulmonary metastasis models were established by tail intravenous injection of 1 × 10^5^ cells diluted in 100 μL PBS (A549-Luc^+^/Le-scrambled infected group, *n* = 6; A549-Luc^+^/Le-shCIRP infected group, *n =* 6). The bioluminescence of pulmonary metastatic tumors was measured by a Xenogen IVIS-200 System (Xenogen, USA). The survival of mice after cell transplantation was recorded and analyzed accordingly. After infection with the indicated lentiviral vectors, A549/H460 tumor xenografts were established by subcutaneously inoculating 1 × 10^6^ cells into 6-week-old female BALB/c nude mice (A549/Le-scrambled infected group, *n* = 9; A549/Le-shCIRP infected group, *n =* 9; A549/Le-shCTNNB1 infected group, *n =* 9; H460/Le-scrambled infected group, *n* = 10; and H460/Le-shCIRP infected group, *n =* 10). Twenty-eight days later, animals were sacrificed to harvest the tumor tissues to weight and perform immunohistochemistry assays as well as hematoxylin and eosin staining. All animals received humane care according to the criteria outlined in the “Guide for the Care and Use of Laboratory Animals” prepared by the National Academy.

### Statistical analysis

The log-rank (Mantel-Cox) test was used to evaluate the statistical significance of the correlation between CIRP or CTNNB1 expression and the overall survival of NSCLC patients. The independent sample T test or nonparametric Mann-Whitney test was used accordingly to study the relationship between CIRP or CTNNB1 expression and other variables. We used the Spearman rank test to analyze correlations between variables. The values of qRT-PCR, the cell growth rate, the cell cycle percentage, aggressive cell numbers, and colony formation are presented as the means ± SD and were compared at a given time point by a two-tailed independent sample T-test. Data were considered to be statistically significant when * *P* < 0.05 and ** *P* < 0.01.

Other methodologies are detailed in the Supplementary information.

## Results

### Elevated expression of CIRP in NSCLC samples correlates with poor prognosis

**Fig. 1:**
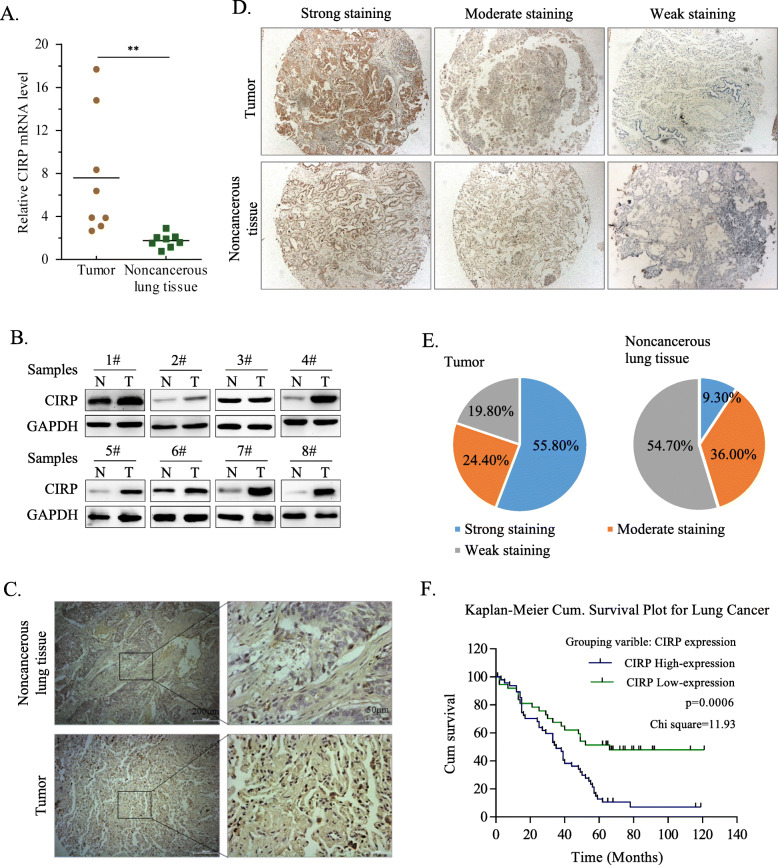
Elevated Expression of CIRP in NSCLC Samples Correlates with Poor Prognosis. (A) mRNA levels of CIRP were quantified in NSCLC samples and their corresponding noncancerous lung tissues as well as normal lung tissues by qRT-PCR (*n =* 8). CIRP expression was normalized to GAPDH expression and each noncancerous lung tissue was used as a control. Data are shown as the mean ± SD (**, *P* < 0.01). (B) The protein level of CIRP was determined by immunoblotting. GAPDH expression served as an endogenous reference. (C) Immunohistochemical analysis of CIRP expression was performed in NSCLC samples and their corresponding noncancerous lung tissues. Representative microphotographs of CIRP expression are displayed. (D) Representative microphotographs showed strong, moderate and weak staining of CIRP in NSCLC tissues and their corresponding noncancerous lung tissues by IHC in a tissue array (magnification × 40). (E) The percentages of strong, moderate and weak CIRP expression in NSCLC samples and corresponding noncancerous lung tissues are shown in pie charts. (F) Kaplan-Meier analysis of the overall survival of NSCLC patients according to the expression level of CIRP protein in NSCLC tissues (CIRP high -expression, *n* = 48 and CIRP low -expression, *n* = 38)

To evaluate the expression of CIRP in NSCLC specimens, we measured the mRNA level of CIRP in eight fresh NSCLC samples and their paired non- NSCLC tissues. Our results revealed that the mRNA level of CIRP in NSCLC tissues was significantly higher than in non-NSCLC tissues (Fig. 1A). The protein level of CIRP was found increased in NSCLC samples by immunoblotting (Fig. 1B). Immunohistochemical staining further showed an elevated expression of CIRP in cancerous tissue. CIRP protein was localized in both the nuclei and plasma of cancer cells (Fig. 1C). These findings suggested that the CIRP expression was obviously upregulated in NSCLC. Subsequently, we investigated the correlation between the aberrant expression of CIRP and prognosis in NSCLC patients. A tissue array containing 86 NSCLC samples and their corresponding noncancerous lung tissues was generated to determine the expression levels of CIRP by immunohistochemical staining. NSCLC and noncancerous samples with strong, moderate or weak CIRP expression are shown by representative images (Fig. 1D). In total, 48 NSCLC samples (55.8%) showed strong CIRP expression, while 21 NSCLC samples (24.4%) and 17 NSCLC samples (19.8%) showed moderate and weak CIRP expression, respectively. In contrast, CIRP was relatively lowly expressed in noncancerous lung tissues. Only 9.3% (8 cases) of noncancerous samples had strong CIRP expression, while the percentages of moderate and weak CIRP expression were 36.0% (31 cases) and 54.7% (47 cases), respectively (Fig. 1E). Further correlation analysis indicated that overexpression of CIRP was correlated with clinicopathological characteristics of patients with NSCLC including the T stage (*P* = 0.039) and an increased incidence of death (*P* = 0.002) as well as lymph node metastasis (*P* = 0.023) (Supplementary Table S1). Moreover, patients demonstrating high expression of CIRP patients had shorter total survival than patients demonstrating low expression of CIRP (*P* = 0.0006) (Fig. 1F).

### CIRP promotes the growth of NSCLC cells

**Fig. 2:**
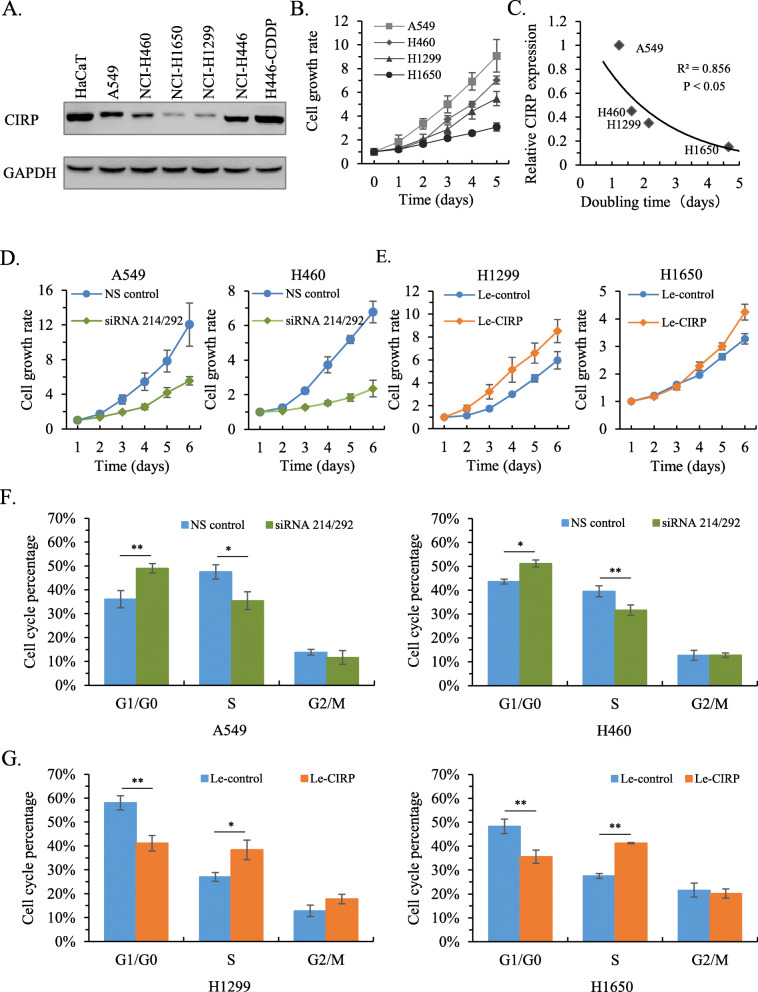
CIRP Regulates the Growth of NSCLC Cells. (A) The protein level of CIRP in lung cancer cell lines was determined by immunoblotting. The HaCaT cell line served as a positive control. (B) Growth rates of NSCLC cell lines with various levels of CIRP expression as determined by the cell proliferation assay. Data were means ± SD from three independent experiments. (C) A significant inverse correlation is shown between CIRP protein levels and the cell doubling time for these NSCLC cell lines. (D) Growth rates of A549 and H460 cells, in which CIRP expression was silenced by siRNA, were determined. Data are the means ± SD from three independent experiments. (E) Growth rates of CIRP stably overexpressing H1299 and H1650 cells were determined. Data are the means ± SD from three independent experiments. (F) Cell cycle analysis was performed in A549 and H460 cells 72 h after transfection with the indicated siRNAs. The average percentages at G1/G0, S and G2/M phases are shown as the means ± SD from three independent experiments (*, *P* < 0.05 and **, *P* < 0.01). (G) Cell cycle analysis was performed in H1299 and H1650 cells stably overexpressing CIRP. The average percentages at G1/G0, S and G2/M phases are shown as the means ± SD from three independent experiments (*, *P <* 0.05 and **, *P <* 0.01)

The protein level of CIRP was evaluated in lung cancer cell lines. The HaCaT cell line served as a positive control because of the validated CIRP expression in our previous study [12]. Relatively high expression levels of CIRP protein were observed in A549, H460 and cisplatin resistant H446 (H446-CDDP) cell lines, but not in H1299 and H1650 cell lines (Fig. 2A). The cells demonstrating high expression of CIRP (A549 and H460) had increased growth rates compared with the cells demonstrating low expression of CIRP (H1299 and H1650) (Fig. 2B). Correlation analysis revealed that the doubling time of growth in these NSCLC cell lines and their CIRP protein expression were inverse correlated (Fig. 2C). These results suggest that CIRP may be involved in the regulation of NSCLC cell growth. To further verify this hypothesis, we synthesized three siRNAs that specifically targeted CIRP mRNA. Our data showed that siRNA 214 and siRNA 292 distinctly inhibited the expression of CIRP, while siRNA 558 had a relatively weak effect of silencing CIRP expression in A549 cells (Supplementary Fig. 1A). By combining siRNA 214 and siRNA 292, the expression of CIRP was robustly knocked down in both A549 and H460 cells (Supplementary Fig. 1B). We found that silencing CIRP inhibited the growth of A549 and H460 cell lines (Fig. 2D). In addition, CIRP-low H1299 and H1650 cells were infected with CIRP-expressing lentiviral vector (Le-CIRP) to investigate its effect on the growth of NSCLC cells. Immunoblotting analysis showed an elevated expression of CIRP in H1299/Le-CIRP and H1650/Le-CIRP cells (Supplementary Fig. 1C). H1299/Le-CIRP and H1650/Le-CIRP cells exhibited higher proliferation rates than H1299/Le-control and H1650/Le-control cells (Fig. 2E). Consistently, cell cycle analysis revealed that silencing CIRP expression in A549 and H460 cells caused G1/GO arrest (Fig. 2F and Supplementary Fig. 2A). The Overexpression of CIRP in H1299 and H1650 cells resulted in accelerated G1/G0 to S transition (Fig. 2G and Supplementary Fig. 2B). Together, these results indicated a growth-promoting role of CIRP in NSCLC cells.

### CIRP promotes the aggressive capability of NSCLC cells

**Fig. 3:**
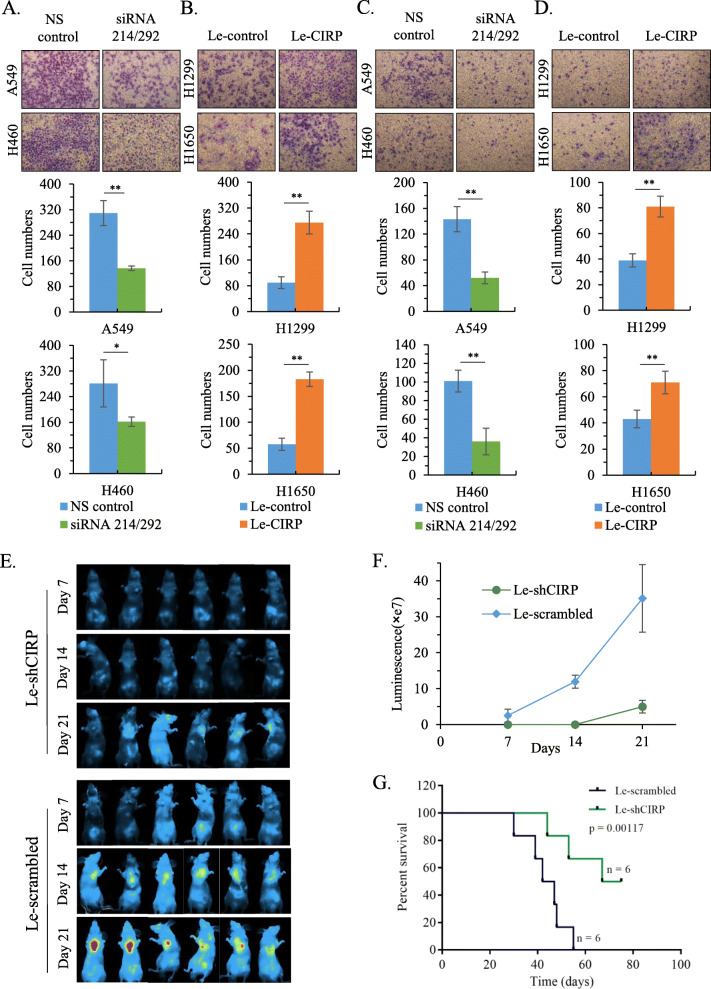
CIRP Promotes the Aggression of NSCLC Cells. (A) Representative microphotographs showing the migration of A549 and H460 cells in which CIRP expression was silenced by siRNA. The migrated cell numbers are shown in histograms. (B) Representative microphotographs showing the migration of CIRP-overexpressing H1299 and H1650 cells. The migrated cell numbers are shown in histograms. (C) Representative microphotographs showing the invasion of A549 and H460 cells in which CIRP expression was silenced by siRNA. The invaded cell numbers are shown in histograms. (D) Representative microphotographs showing the invasion of CIRP-overexpressing H1299 and H1650 cells. The invaded cell numbers are shown in histograms. All data are the means ± SD from three independent experiments (**, *P <* 0.01). (E) Luciferase images of mice at 7-, 14-, and 21-days post-tail intravenous injection with Le-shCIRP or Le-Scrambled -infected A549-Luc^+^ (*n* = 6, per group). (F) The average bioluminescence signals in the Le-shCIRP group at different time points are compared with those in the A549-Luc^+^ cells transduced with the Le-Scrambled group (mean ± SD; *n =* 6, **, *P <* 0.01). (G) Survival analysis of mice implanted with A549-Luc^+^ cells with or without CIRP knockdown

To further study the biological function of CIRP in NSCLC cells, we analyzed the effect of CIRP on the migration and invasion ability of NSCLC cells. Our data showed that silencing CIRP with siRNA214/292 inhibited the migration of A549 and H460 cells compared with the NS control siRNA (Fig. 3A). Le-CIRP-infected H1299 and H1650 cells exhibited higher migration ability than Le-control-infected cells (Fig. 3B). Consistently, we also found that silencing CIRP inhibited the invasion capability of A549 and H460 cell lines (Fig. 3C), while Le-CIRP-infected H1299 and H1650 cells displayed increased invasion capacity in comparison with Le-control-infected cells (Fig. 3D). In addition, A549 and H460 cells with stable overexpression of CIRP were established (Supplementary Fig. 3A). We found that elevated CIRP expression promoted both the migration and invasion capability of A549 and H460 cells (Supplementary Fig. 3B and C). These results suggested a metastasis-promoting role of CIRP in NSCLC cells. To further confirm this observation, we constructed a lentiviral vector carrying short hairpin RNA (shRNA) that specifically knocked down CIRP expression (Le-shCIRP) and a scrambled control lentiviral vector (Le-scrambled) [13]. We found that infection of A549 and H460 cells with Le-shCIRP at a multiplicity of infection (MOI) of 10 inhibited CIRP expression more efficiently than infection of cells with Le-shCIRP at an MOI of 1 (Supplementary Fig. 3D and E). Thus, we used a MOI of 10 in the following experiments. The role of CIRP in the metastasis of NSCLC cells was also investigated in an animal model. We infected A549 stably expressing luciferase cells (A549-Luc^+^) with Le-shCIRP or Le-scrambled. At 72 h after infection, A549-Luc^+^ cells (1 × 10^5^) were intravenously injected via the tail vein to establish a metastatic model. Our data showed that CIRP downregulation resulted in a significant reduction in pulmonary space-occupying lesions by bioluminescence imaging (Fig. 3E) and quantification of the luciferase activity at different time points (Fig. 3F). Compared with nude mice injected with Le-scrambled A549-Luc^+^ cells, the mice bearing tumors derived from CIRP-depleted A549-Luc^+^ cells showed significantly prolonged survival as assessed by Kaplan–Meier survival curves (*P* = 0.00117) (Fig. 3G).

### CIRP promotes the tumorigenesis of NSCLC cells

**Fig. 4:**
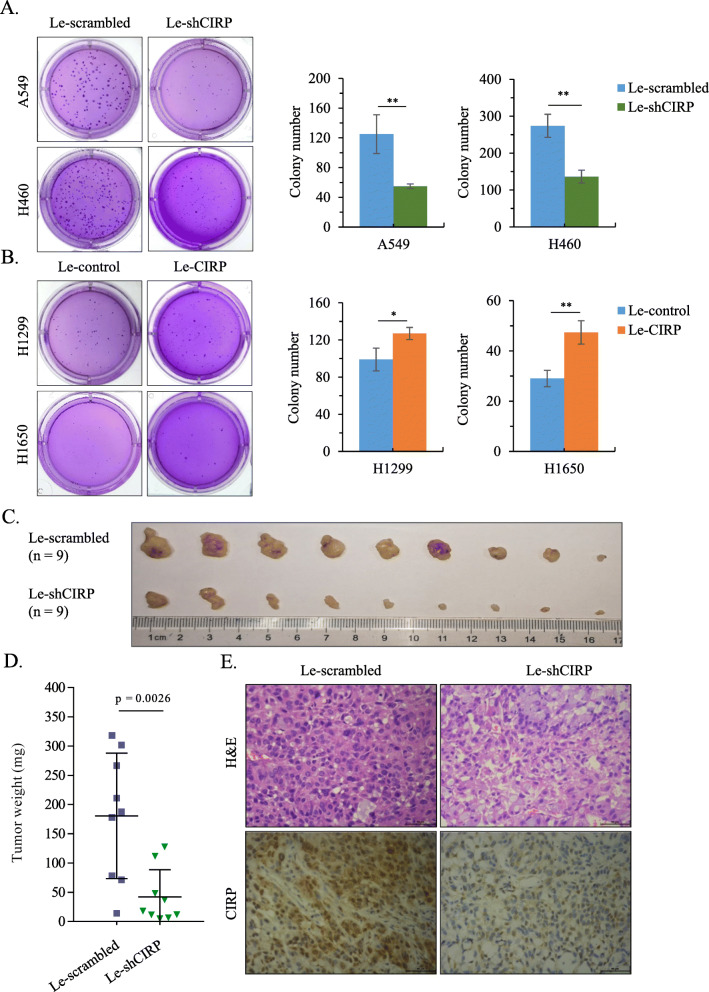
CIRP Promotes the Tumorigenesis of NSCLC Cells. (A) Representative photographs showing the effect of silencing CIRP on the colony formation of A549 and H460 cells. The clone numbers are shown in histograms representing the means ± SD from three independent experiments (**, *P* < 0.01). (B) Representative photographs showing the effect of CIRP overexpression on the colony formation of H1299 and H1650 cells. The clone numbers are shown in histograms representing the means ± SD from three independent experiments (*, *P <* 0.05 and **, *P <* 0.01). (C) A549 cells were subcutaneously inoculated into BALB/c nude mice after infection with Le-scrambled or Le-shCIRP at an MOI of 10 (*n* = 9 for each group). Twenty-eight days later tumors were harvested. (D) The weight of established tumors was measured and is shown in a scatter plot. (E) Immunohistochemical analysis of CIRP expression was performed on these xenografts. Representative images are shown (magnification × 200)

We found that the efficiency of colony formation was decreased when A549 and H460 cells were infected with Le-shCIRP (Fig. 4A), while Le-CIRP-infected H1299 and H1650 cells displayed an increased colony forming capacity in comparison with their Le-control-infected counterparts (Fig. 4B). The role of CIRP in NSCLC cell tumor formation was also investigated in animal models. Our data showed that the tumors derived from Le-shCIRP-infected A549 and H460 cells were much smaller than those derived from Le-scrambled infected A549 and H460 cells (Fig. 4C and Supplementary Fig. 4A). The average weight of tumors was significantly lower in the Le-shCIRP-infected group than in the Le-scrambled infected group (*P* = 0.0026 and *P* = 0.005) (Fig. 4D and Supplementary Fig. 4B). Immunohistochemical staining analysis revealed extensive expression of CIRP in tumors from the Le-scrambled-infected group, whereas CIRP expression was barely detectable in the formed tumors from the Le-shCIRP-infected group (Fig. 4E and Supplementary Fig. 4C). These data indicated that CIRP regulates tumor formation both in vitro and in vivo.

### CIRP regulates the Wnt/β-catenin signaling pathway in NSCLC cells

**Fig. 5:**
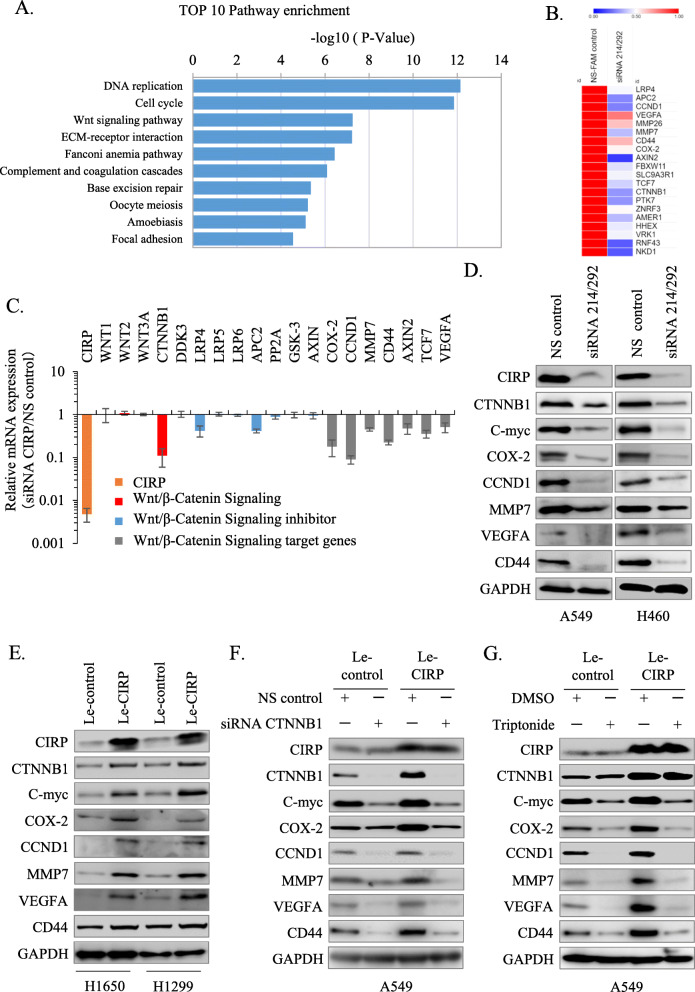
CIRP Regulates the Wnt/β-catenin Signaling Pathway in NSCLC Cells. (A) The top 10 enriched pathways affected by CIRP downregulation in A549 cells are shown. (B) Heat map showing the mRNA levels of genes involved in the Wnt signaling pathway in CIRP silenced A549 cells. Red and blue indicate higher and lower gene expression, respectively, and the magnitude of expression differences is displayed by the color intensity. (C) qRT-PCR analysis the of mRNA levels of Wnt/β-catenin signaling pathway related genes was performed in CIRP-silenced A549 cells. Data were normalized to the levels in NS control A549 cells, and are shown as the means ± SD from three independent experiments. (D) Protein levels of the indicated genes in CIRP silenced A549 and H460 cells were determined by immunoblotting. (E) Protein levels of the indicated genes in CIRP-overexpressing H1299 and H1650 cells were determined by immunoblotting. (F) Protein levels of the indicated genes in CIRP-overexpressing and control A549 cells cotransfected with control or CTNNB1 siRNA for 48 h. (G) Protein levels of the indicated genes in CIRP-overexpressing and control A549 cells treated with IC_50_ of Triptonide for 48 h

To elucidate the mechanisms by which CIRP promotes the proliferation, metastasis and tumor formation of NSCLC cells. We examined global gene expression profiles in A549 cells after transfection with siRNA214/292 against CIRP and control siRNA by cDNA microarray. By analysis of the data, we found that 1194 genes were upregulated and 585 genes were downregulated with a 2-fold or higher change in their expression when CIRP was knocked down. Kyoto Encyclopedia of Genes and Genomes (KEGG) systematic analysis [26] revealed that these genes were enriched in the categories of various cell processes, including DNA replication, cell cycle, Wnt signaling pathway, ECM-receptor interaction, Fanconi anemia pathway, TNF signaling pathway, MAPK signaling pathway and so on (Fig. 5A). Considering the relatively high enrichment of Wnt signaling and its important role in the development of cancer, we next focused on investigating the relationship between CIRP and the Wnt signaling pathway. According to the sequencing data, 20 candidate genes were downregulated by silencing CIRP expression (Fig. 5B). Further qRT-PCR analysis indicated that among these Wnt/β-catenin signaling promoting genes, only CTNNB1 was decreased in CIRP-silenced A549 cells compared with the control A549 cells; genes inhibiting Wnt/β-catenin signaling such as LRP4 and APC2 were also downregulated. Meanwhile, the mRNA abundance of the target genes of Wnt/β-Catenin signaling, including COX-2, CCND1, MMP7, CD44, AXIN2, TCF7 and VEGFA, was reduced when CIRP expression was depressed in A549 cells (Fig. 5C). Immunoblotting analysis further confirmed that the protein levels of CTNNB1, C-myc, COX-2, CCND1, MMP7, VEGFA and CD44 were reduced in CIRP-silenced A549 and H460 cells, as compared with NS control cells (Fig. 5D). In contrast, the infection of, H1650, H1299 A549 and H460 cells with Le-CIRP resulted in increased protein levels of these genes compared with Le-control infected parental cells (Fig. 5E and Supplementary Fig. 5A). Further, we found that silencing of CTNNB1 could nullify the upregulation of those Wnt target genes in CIRP overexpression A549 cells, and the expression of CIRP was not affected (Fig. 5F), while silencing of CIRP had no significant effect on the expression of those Wnt target genes except for CCND1 and C-myc in CTNNB1 overexpression A549 cells (Supplementary Fig. 5B). In addition, a specific Wnt signaling inhibitor triptonide, which blocks the C-terminal transactivation domain of the CTNNB1 protein [27], was used to determine the role of CIRP activity in regulation of Wnt signaling. Cell viability analysis showed that triptonide inhibited A549 cells (Le-control and Le-CIRP) growth in a concentration dependent manner with an IC_50_ at the concentration of approximate 10 nM (Supplementary Fig. 5C). Consistent with the results of silencing CTNNB1, IC_50_ of triptonide treatment also abolished the upregulation of those Wnt target genes in CIRP overexpression A549 cells (Fig. 5G). These findings suggested that CIRP might mediate the canonical Wnt pathway by regulating the expression of CTNNB1.

### Overexpression of CTNNB1 in Cancer cells correlates with poor prognosis of patients with NSCLC

To investigate whether the aberrant expression of CTNNB1 predicts prognosis in NSCLC patients, we used the same tissue array to determine CTNNB1 expression levels by immunohistochemical staining. Our data showed that strong, moderate or weak CTNNB1 expression was observed in both NSCLC and noncancerous tissues (Supplementary Fig. 6A). A total of 45.3% of NSCLC samples showed strong CTNNB1 expression, while the percentages of moderate and weak CIRP expression were 36.1 and 18.6%, respectively (Supplementary Fig. 6B). In contrast, CTNNB1 expression was relatively low in noncancerous lung tissues. Approximately 22.1% of noncancerous samples had strong CTNNB1 expression, while the percentages of moderate and weak CTNNB1 expression were 62.8 and 15.1%, respectively (Supplementary Fig. 6C). Clinicopathological analysis showed that elevated expression of CTNNB1 in NSCLC was correlated with the tumor stage (*P* = 0.026), an increased incidence of death (*P* = 0.024), and lymph node metastasis (*P* = 0.007) (Supplementary Table S1). Similar to CIRP, patients with high CTNNB1 expression had shorter total survival than patients with low CTNNB1 expression (*P* = 0.0063) (Supplementary Fig. 6D).

### Knockdown of CTNNB1 suppressed the tumorigenesis of NSCLC cells

Next, we investigated the role of CTNNB1 in NSCLC cell tumor formation in an animal model. We constructed a lentiviral vector carrying shRNA that specifically knocked down CTNNB1 expression (Le-shCTNNB1) [28]. Le-shCTNNB1 infected A549 cells at an MOI of 10 formed small tumors in 78% of nude mice. In contrast, Le-scrambled infected cells formed tumors in 100% of nude mice, and the volumes of tumors were much larger (Supplementary Fig. 7A). The average weight of tumors was significantly lower in the Le-shCTNNB1-infected group than in the Le-scrambled infected group (*P* = 0.0008) (Supplementary Fig. 7B). By immunohistochemical staining analysis, we observed extensive expression of CTNNB1 in tumors derived from the Le-scrambled-infected group, while CTNNB1 expression was quite weak in tumors derived from the Le-shCTNNB1-infected group (Supplementary Fig. 7C). These data showed that CTNNB1 silencing suppressed NSCLC formation in vivo.

### Association of CIRP and CTNNB1 in NSCLC tissues

**Fig. 6:**
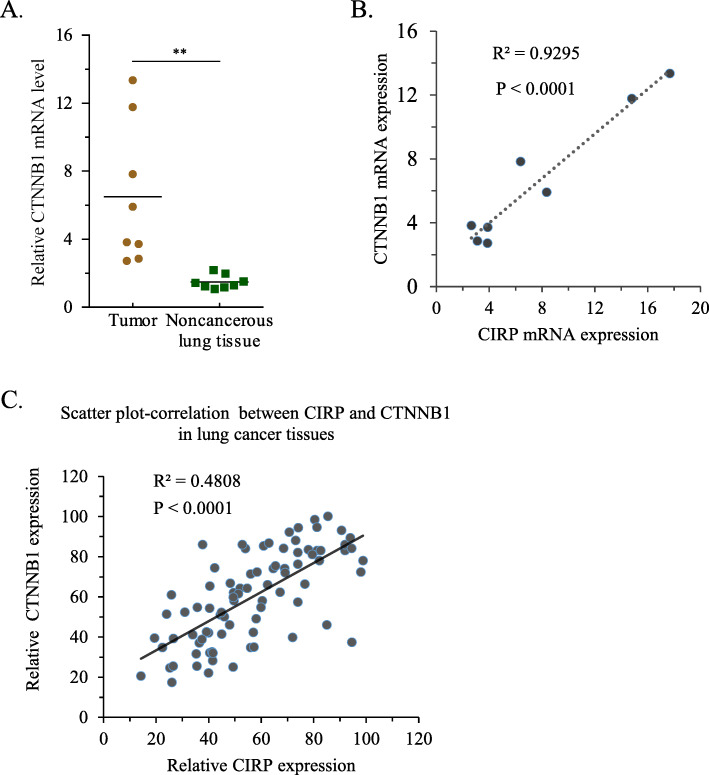
Expression of CIRP and CTNNB1 is Positively Correlated in NSCLC Tissues. (A) mRNA levels of CTNNB1 were quantified in NSCLC samples and their corresponding noncancerous lung tissues as well as normal lung tissues by qRT-PCR (*n* = 8). CTNNB1 expression was normalized to GAPDH expression and each noncancerous lung tissue was used as a control. Data are shown as the mean ± SD (**, *P <* 0.01). (B) The correlation between CIRP mRNA and CTNNB1 mRNA expression in NSCLC tissues. (C) The correlation between the protein levels of CIRP and CTNNB1 in NSCLC tissues is shown in a scatter plot

To further clarify the regulatory relationship between CIRP and CTNNB1, we next detected the mRNA levels of CTNNB1 in eight fresh NSCLC samples and paired noncancerous tissues. Similar to CIRP expression, the mRNA abundance of CTNNB1 was significantly higher in NSCLC tissues than in noncancerous tissues (Fig. 6A). By analyzing the mRNA abundance of CIRP and CTNNB1, we found that CIRP and CTNNB1 were positively correlated in these cancer samples (Fig. 6B). In our research, we were surprised to find that the expression levels of CIRP and CTNNB1 were very similar according to Spearman’s correlation. By analyzing the immunohistochemical staining of CIRP and CTNNB1 in a tissue array, we found that CTNNB1 expression was positively associated with CIRP expression (*P* < 0.001) (Fig. 6C). Although this correlation is statistically significant, it is worth pondering whether CIRP directly regulates CTNNB1 in NSCLC cells and what mechanism underlies this regulation.

### CIRP increases CTNNB1 mRNA stability and protein translation in NSCLC cells

**Fig. 7:**
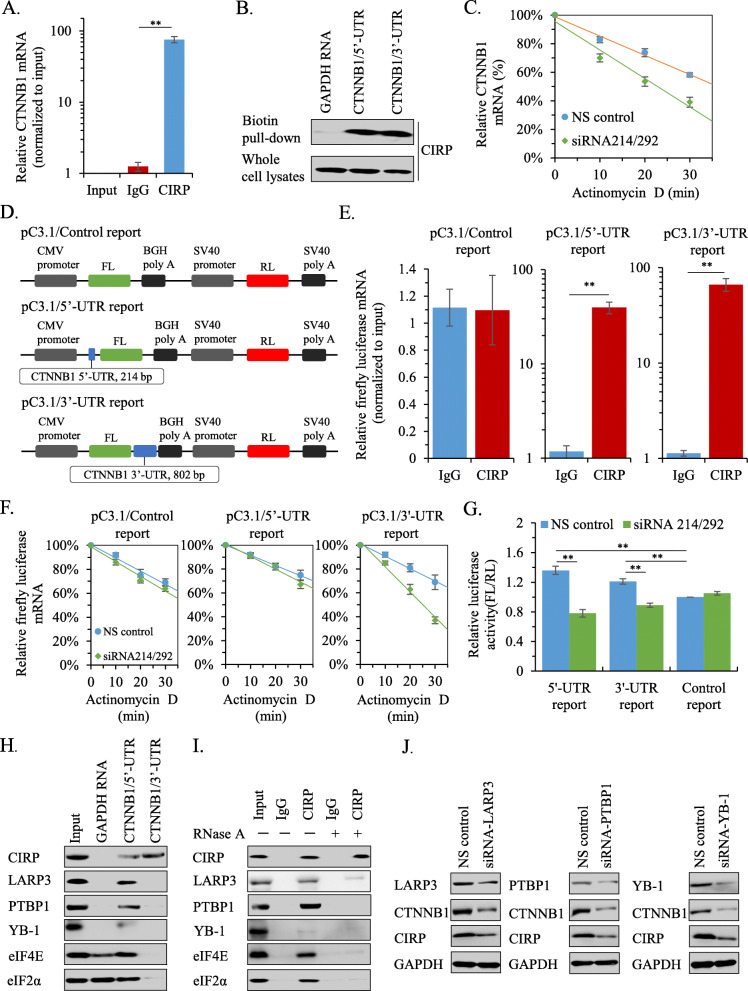
CIRP Post-transcriptionally Regulates the Expression of CTNNB1. (A) mRNA levels of CTNNB1 in the RIP assay were quantified by qRT-PCR. The level in the input group was used as a control. Data are the means ± SD from three independent experiments (**, *P <* 0.01). (B) Immunoblotting detected the pulled CIRP protein in the biotin pull-down assay. Transcripts of GAPDH mRNA were used as a negative control. (C) Degradation of CTNNB1 mRNA was analyzed in A549 cells transfected with CIRP or control siRNAs by qRT-PCR. Data are the means ± SD from three independent experiments. (D) Schematic diagrams of the indicated luciferase reporter vectors. (E) mRNA levels of firefly luciferase in the RIP assay were quantified by qRT-PCR. Data were normalized to the input group and are presented as the means ± SD from three independent experiments (**, *P <* 0.01). (F) Degradation of firefly luciferase mRNA was analyzed by qRT-PCR in A549 cells cotransfected with luciferase reporter vectors and CIRP or control siRNAs. Data are shown as the means ± SD from three independent experiments. (G) Luciferase expression was measured in A549 cells cotransfected with CIRP or control siRNAs and luciferase vectors. The fold changes of relative luciferase activity in CIRP siRNA with indicated luciferase reporter transfected cells were normalized to NS control with pC3.1/control reporter transfected cells. Data are the means ± SD from three independent experiments (**, *P <* 0.01). (H) Immunoblotting detected the indicated proteins in the biotin pull-down assay. Twenty micrograms of whole cell lysates of A549 cells (input) served as a positive control. (I) Immunoblotting analysis of the indicated proteins in input and elute upon copurification with anti-CIRP or IgG from A549 cells. Immunopurifications were performed in the absence (−) or presence (+) of 10 mg/mL RNase A. (J) Immunoblotting analysis of CIRP and CTNNB1 expression in the indicated IRES trans-acting factor silenced A549 cells was performed

To elucidate the mechanism by which CIRP regulates CTNNB1 expression in NSCLC cells, we implemented RNA-IP experiments. Our data showed that both pairs of CTNNB1 mRNA detection primers could successfully amplify the target fragments in the anti-CIRP IP group as well as in the input positive control group (Supplementary Fig. 8A). In addition, qRT-PCR analysis revealed that the anti-CIRP IP group successfully enriched CTNNB1 mRNA by approximately 76-fold compared with the input control group (Fig. 7A). Furthermore, biotin pull-down assay was performed to further confirm the interaction of CIRP and CTNNB1 mRNA. Our data showed that a large amount of CIRP protein was associated with the 5′-UTR (untranslated region) and 3′-UTR of CTNNB1 mRNA, nevertheless, the negative control transcripts failed to pull down CIRP (Fig. 7B). Next, RNA decay analyses were performed in the presence of actinomycin D (a transcriptional inhibitor, 15 μg/mL). Our results showed that knockdown of CIRP by siRNA led to the accelerated decay of CTNNB1 mRNA (Fig. 7C).

To further explore the combination of CIRP with the 5′-UTR and 3′-UTR of CTNNB1 mRNA, we constructed a pC3.1/control reporter vector by replacing the original neomycin gene with the Renilla luciferase (RL) coding DNA fragment and inserting the firefly luciferase (FL) coding DNA fragment into the multiple cloning site (MCS) of pcDNA3.1. Thereafter, the 5′-UTR and 3′-UTR coding DNA were cloned into the flank of the FL coding region to form pC3.1/5′-UTR reporter and pC3.1/3′-UTR reporter vectors (Fig. 7D). Moreover, a pC3.1/CDS reporter vector was also created by fusion expression of FL-CTNNB1 protein (Supplementary Fig. 8B). After the transfection of each reporter vector into A549 cells for 72 h, RNA-IP experiments were performed. Our results showed that the anti-CIRP IP groups successfully enriched FL mRNA in pC3.1/5′-UTR reporter- and pC3.1/3′-UTR reporter-transfected cells, but not in pC3.1/control reporter- and pC3.1/CDS reporter-transfected cells (Fig. 7E and Supplementary Fig. 8C). These data suggested that CIRP could directly bind to the 5′-UTR and 3′-UTR of CTNNB1 mRNA in NSCLC cells. Then, RNA decay analyses were performed in A549 cells cotransfected with each reporter vector and CIRP siRNA or NS siRNA. qRT-PCR analysis revealed that silencing CIRP expression resulted in the accelerated degradation of CTNNB1 3′-UTR-fused FL mRNA, while the decay of FL control mRNA and CTNNB1 5′-UTR or CTNNB1 CDS fused FL mRNA was not affected by CIRP knockdown (Fig. 7F and Supplementary Fig. 8D). In addition, a luciferase activity assay revealed that CIRP silencing resulted in decreased relative luciferase activity in pC3.1/5′-UTR reporter- and pC3.1/5′-UTR reporter-transfected A549 cells, whereas no significant inhibitory effect was observed in A549 cells transfected with pC3.1/control reporter or pC3.1/CDS reporter vectors when CIRP expression was silenced (Fig. 7G and Supplementary Fig. 8E). It is worth noting that without changing CIRP expression, the relative luciferase activity in A549 cells transfected with pC3.1/5′-UTR reporter or pC3.1/3′-UTR reporter vectors was higher than that in pC3.1/CDS reporter-transfected cells (Fig. 7G). These results suggested that CIRP bound to the 3′-UTR of CTNNB1 mRNA to increase its stability and facilitate its translation in NSCLC cells.

### CIRP promotes IRES-dependent translation of CTNNB1 in NSCLC cells

Our data also indicated that CIRP binds to the 5′-UTR of CTNNB1 mRNA to promote protein translation. Nevertheless, the exact molecular mechanism has not been illustrated. It has been reported that the 5′-UTR of CTNNB1 mRNA contains an internal ribosome entry segment (IRES) that regulates its translation [29]. Given that CIRP is a known RNA-binding protein, and its main role in the cytoplasm is to bind specific mRNAs (IRES-containing mRNAs) to facilitate their translation upon stress [7], we wondered whether the cytoplasmic CIRP of NSCLC cells would also promote the IRES-dependent translation of CTNNB1. To verify our conjecture, we performed a biotin pull-down assay in A549 cells. We found that the 5′-UTR of CTNNB1 mRNA was able to pull down the proteins that were closely related to the IRES-dependent translation process, such as LARP3, PTBP1, YB-1, eIF4E and eIF2α (Fig. 7H). Further immunopurification assays demonstrated that all of the above indicated that proteins were copurified with CIRP from the cytoplasmic extracts of A549 cells (Fig. 7I, RNaseA-). However, copurification was abolished or severely reduced in the presence of RNaseA (Fig. 7I, RNaseA+). Additionally, knockdown of the IRES trans-acting factors (ITAFs: LARP3, PTBP1 and YB-1) separately with their siRNAs resulted in reductions of CTNNB1 protein in A549 cells, while to our surprise, the expression of CIRP was also reduced (Fig. 7J). These findings suggested that CIRP directly interacted with CTNNB1 by binding to its 5′-UTR to promote IRES-dependent protein translation in NSCLC cells.

## Discussion

CIRP has been linked to tumor growth and metastasis as an oncogene in several types of human cancer [12, 21, 30–32]. However, the exact function(s) of CIRP in the regulation of critical cellular activities and progression of NSCLC remain unclear. We demonstrated here that CIRP was overexpressed in 55.8% of NSCLC tissues and was correlated with more frequent lymph node metastasis and shorter time of overall survival, suggesting that the CIRP expression level is associated with prognosis. Our data also demonstrated that there is a positive correlation between CIRP and CTNNB1 expression in NSCLC cells, and CIRP regulates CTNNB1 post-transcriptionally by binding to the 5′-UTR and 3′-UTR of CTBBN1 mRNA. In addition to enhancing CTNNB1 mRNA stability, CIRP could also facilitate IRES-dependent translation to promote CTNNB1 accumulation in NSCLC cells.

We observed a correlation between the CIRP expression level and cell growth rate in lung cancer cells. Knockdown of CIRP in NSCLC cells with high CIRP expression suppressed cell growth, whereas overexpression of CIRP in NSCLC cells with low CIRP expression improved their proliferation. Moreover, knockdown of CIRP in NSCLC cells significantly suppressed their tumorigenicity both in vitro and in vivo. Consistent with our findings in NSCLC, CIRP has also been shown to contribute to cell growth in other tumors. Lu et al. demonstrated that CIRP is required for the HIF-1α-induced proliferation of human bladder cancer cells [21]. Zhou et al. showed that silencing CIRP with siRNA inhibited the proliferation of the 786–0 cells and enhanced their chemosensitivity [32]. This function of CIRP has also been reported in UVB-induced skin carcinogenesis [13]. Our data that knockdown of CIRP in NSCLC cells with high CIRP expression induced cell cycle blockade at the G1/S checkpoint, while overexpressing CIRP in NSCLC cells with low CIRP expression accelerated the G1/G0 to S transition further supported the proliferation-promoting effect of CIRP in NSCLC. The CCND1 gene encodes a well-known G1/S-specific cyclin (Cyclin D1) that promotes the transition of the cell cycle from G1 to S phase [33]. In addition, the proliferation-promoting function of CIRP was further verified by our finding that the expression of CCND1 was positively correlated with CIRP expression.

In addition to its proliferation-promoting effects, CIRP has been shown to promote tumor metastasis in several types of cancers. Wang et al. demonstrated that CIRP expression was significantly upregulated in pituitary adenoma and contributed to tumor invasion [34]. Lee et al. reported that CRIP contributed to TGF-β1-induced EMT in human lung carcinoma A549 and hepatocellular carcinoma Huh7 cells [30]. Here our clinicopathological analysis showed that CIRP overexpression was correlated with increased incidence of lymph node metastasis in NSCLC. Our in vitro experimental data demonstrated that knockdown of CIRP in NSCLC cells with high CIRP expression suppressed cell migration and invasion, and accordantly overexpressing CIRP in NSCLC cells with low CIRP expression enhanced cell migration and invasion. Moreover, knockdown of CIRP in A549-Luc^+^ cells reduced the numbers of pulmonary space-occupying lesions when these cells were administered to mice via tail vein injection. Although all of these studies support an aggression-promoting function of CIRP in tumors, there is one study in which CIRP has been shown to suppress cell migration in BEV-treated glioma cells [35]. This discrepancy may be due to cell type differences, however future studies are needed to explore the distinct functions of CIRP in different diseases.

The Wnt/β-catenin pathway plays a central role in the development of many human cancers by regulating cell proliferation, migration, and invasion [36–38]. As the core factor in the canonical Wnt/β-catenin pathway, CTNNB1 can combine with E-cadherin to form a cadherin/catenin complex and maintain cell-cell adhesion [39]. When the Wnt signaling pathway is activated, CTNNB1 accumulation will occur in the nucleoplasm of tumor cells, leading to the loss of epithelial structural integrity and increased tumor invasion and metastasis [40, 41]. In addition, CTNNB1 can act as a signal transduction molecule and its activation significantly stimulates the production of VEGF, upregulates the expression of MMPs to enhance the degradation of extracellular matrix (ECM), and thus promotes tumor cell growth, invasion and metastasis [42]. CTNNB1 has been demonstrated to be oncogenic in several tumor types and its expression is regulated by multiple Wnt molecules [43–45]. Previous studies have shown that Wnt/β-catenin signaling promoters such as Wnt1, Wnt2 and Wnt3a are associated with tumor proliferation and angiogenesis in NSCLC [46–49]. Chuan et al. have demonstrated that the transcription of CTNNB1 is directly regulated by Wnt-1 in oral cancer [50]. A very recent study also reported that nuclear abnormal expression of CTNNB1 is associated with mutations of the CTNNB1 gene in lung cancer [51]. Wu et al. have shown that the polyubiquitination and degradation of TRAF6 induces the accumulation of CTNNB1, which contributes to aberrant activation of Wnt/β-catenin signaling and thereby promotes the epithelial-mesenchymal transition and metastasis (EMT) of colorectal cancer (CRC )[52]. Here our data show that increased CTNNB1 expression in tumor tissues is correlated with poor prognosis in NSCLC patients. Moreover, the expression of CTNNB1 and the downstream target genes of Wnt/β-catenin signaling are positively correlated with CIRP expression in NSCLC cells.

CIRP belongs to the family of RNA binding proteins and can specifically bind the 3′-UTRs of mRNAs to stabilize target mRNAs and prolong their translation lifetimes. In human bladder cancer cells, CIRP has been shown to bind the 3′-UTR of HIF-1α mRNA, thereby increasing its stability and extending its translation [21]. Yang et al. reported that hypothermia induces an airway inflammatory response through CIRP-mediated increases in pro-inflammatory cytokine mRNA stability and protein translation [53]. Consistent with these findings, our data demonstrate that the binding of the CIRP protein to the 3′-UTR of CTNNB1 mRNA enhances mRNA stability and increases protein translation. It is worth pointing out that the CIRP protein can also bind the 5′-UTR of CTNNB1 mRNA and this binding enhances protein translation, but has no significant effect on mRNA stability. To further determine how CIRP CTNNB1 mRNA 5′-UTR binding promotes CTNNB1 translation, we investigated the interaction of CIRP with other translation-associated factors. Our data show that CIRP can bind to translation initiation complex-related proteins (eIF4E and eIF2α) and IRES trans-acting factors (LARP3, PTBP1 and YB-1). Since CTNNB1 mRNA contains an IRES sequence in its 5′-UTR [29], we hypothesized that CIRP might bind to the 5′-UTR of CTNNB1 to promote IRES-dependent translation in NSCLC cells. We conducted immunopurification assays and verified our speculation. Recently, Liu et al. demonstrated that m6A mRNA methylation significantly contributes to the expression and stability of CTNNB1 in hepatoblastoma [54]. Whether and how CIRP interacts with m6A methylation to regulate CTNNB1 mRNA stability is an important question we will answer in future studies. Interestingly, our data show that silencing CIRP could downregulate the expression of CCND1 and C-myc in CTNNB1-overexpressing A549 cells, and CIRP is downregulated in A549 cells when we knocked down the IRES trans-acting factors LARP3, PTBP1 or YB-1 with siRNAs. One possible explanation is that CCND1, C-myc and CIRP mRNA also contain an IRES sequence, and their translation is also partly subject to an IRES-dependent translation process [55–57].

## Conclusions

**Fig. 8:**
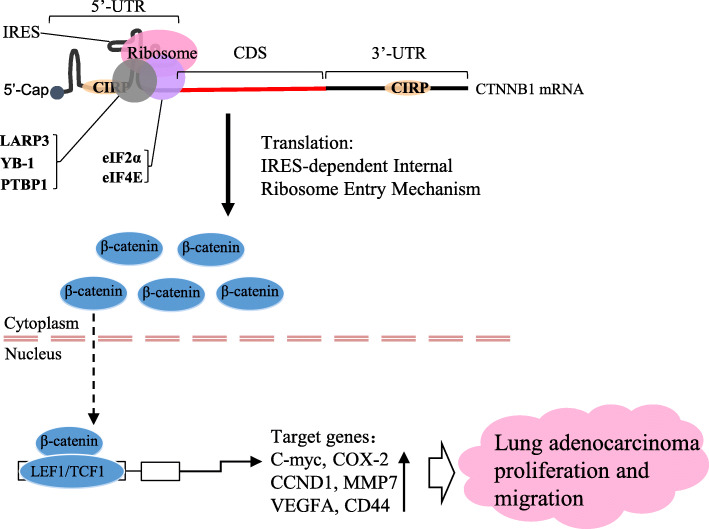
A mechanism of the CIRP-enhanced Wnt/β-Catenin Signaling Pathway. Abnormally increased cytoplasmic CIRP in NSCLC cells promotes CTNNB1 protein expression through post-transcriptional regulation. That is, it binds to the 5′-UTR and 3′-UTR of CTNNB1 mRNA to increase its mRNA stability and facilitate its IRES-dependent translation. The elevated CTNNB1 protein persistently activates the Wnt/β-catenin signaling pathway which results in an increase in downstream oncogene expression and promotes NSCLC progression

In summary, as shown in the mechanistic diagram (Fig. 8), our study demonstrates that CIRP acts as a tumor-promoting gene to promote cell proliferation and aggression in NSCLC both in vitro and in vivo. Mechanistically, cytoplasmic CIRP increases CTNNB1 expression by binding to the 5′-UTR and 3′-UTR of CTNNB1 mRNA to increase its mRNA stability and facilitate its IRES-dependent translation in NSCLC cells. As a result, elevated CTNNB1 expression continuously activates the Wnt/β-catenin signaling pathway, thereby promoting downstream gene expression and tumor progression. Our data suggest that CIRP may be a useful prognostic marker and a potential therapeutic target for NSCLC.

## Supplementary Information

**Additional file 1.**

**Additional file 2.**

## Data Availability

All data generated or analyzed during this study are included in this published article [and its supplementary information files].

## Abbreviations

CIRP: Cold-inducible RNA binding protein
NSCLC: Non-small cell lung cancer
RRM: RNA recognition motif
eCIRP: Extracellular CIRP
DAMP: Damaged-associated molecular pattern
TNF-α: Tumor necrosis factor-alpha
ERK1/2: Extracellular signal-regulated kinase 1/2
HIF-1: Hypoxia inducible transcriptional factor-1
VEGF: Vascular endothelial growth factor
RL: Renilla luciferase
FL: Firefly luciferase
IRES: Internal ribosome entry segment
ITAFs: IRES trans-acting factors
UTR: Untranslated region
ECM: Extracellular matrix
EMT: Epithelial-mesenchymal transition and metastasis
CRC: Colorectal cancer
