# Supplementary material for: CIRP promotes the progression of non-small cell lung cancer through activation of Wnt/β-catenin signaling via CTNNB1
Source: J Exp Clin Cancer Res. 2021 Aug 31;40:275. doi: 10.1186/s13046-021-02080-9 (PMC8406911; doi:10.1186/s13046-021-02080-9)
Supplement: Supplementary file 1 — Additional file 1. [file 13046_2021_2080_MOESM1_ESM.docx]

**CIRP promotes the progression of non-small cell lung cancer through activation of Wnt/β-catenin signaling via CTNNB1**

Yi Liao, Jianguo Feng, Weichao Sun, Chao Wu, Jingyao Li, Tao Jing, Yuteng Liang, Yonghui Qian, Wenlan Liu, Haidong Wang

**Supplementary Materials and Methods**

**Immunoblotting.** Cells were lysed in ice-cold RIPA buffer with a proteinase inhibitor cock-tail (P8340, Sigma-Aldrich, MO, USA). Proteins were resolved by Precast-Gel (PG01210-S, Solarbio, Beijing, China) and transferred to nitrocellulose membranes (P-N66485, Solarbio, Beijing, China). After blocking with 5% fat-free milk for 1 h, the membrane was incubated overnight at 4℃ with primary antibody, which was followed by incubation with a corresponding horseradish peroxidase-conjugated secondary antibody. Membranes were developed using SuperSignal™ West Pico PLUS Chemiluminescent Substrate ( #34580, Thermo Scientific, MA, USA). The primary antibodies and secondary antibodies are listed in **Supplementary Table S2.**

**Total RNA isolation and Quantitative RT-PCR (****qRT-PCR).** Total RNA was harvested from cells and human cancerous or noncancerous samples with Trizol solution (#15596026, Thermo Scientific, MA, USA). cDNA was obtained using SuperScript™ III Reverse Transcriptase (#18080093, Thermo Scientific, MA, USA) according to the manufacturer’s instructions. qRT-PCR was performed using SYBR® Premix Ex Taq™ II (RR820A, Takara, Japan) on CFX96™ Real-Time PCR Detection System (Bio-Rad, CA, USA) as follows: 95 ℃ for 30 s, 40 cycles at 95 ℃ for 5 s, 60 ℃ for 30 s. Fold enrichment was calculated with the −ΔΔCt method relative to GAPDH. The sequences for primers involved in this study are listed in **Supplementary Table S3**.

**Construction of plasmid and viral vectors.** The viral vectors for stable overexpression and transient silencing of CIRP, as well as the control viral vectors were established in our previous study[[1](#_ENREF_1)]. Human CTNNB1 cDNA was cloned into pLenti-CMV-GFP-Puro (plasmid #17448, Addgene) between BamHI and SalI restriction sites to form the overexpression lentiviral vector pLenti-CMV-CTNNB1.The lentiviral shRNA construct for silencing of CTNNB1 (pLKO.1-CTNNB1-shRNA) was constructed by inserting the shRNA coding DNA fragment into plasmid pLKO.1-TRC cloning vector (Addgene plasmid, #10878) between Age I and EcoRI restriction sites. The shRNA sequence for targeting CTNNB1 was reported in Li. et, al’s study[[2](#_ENREF_2)]. Then, pLKO.1-CTNNB1-shRNA as well as lentiviral helper vectors pCMV-VSV-G plasmid (Addgene plasmid, #8454) and pCMV-dR8.2 dvpr plasmid (Addgene plasmid, #8455) were co-transfected into HEK-293T cells for 72 hours to harvest the lentiviral particles. For Luciferase reporters, the neomycin coding DNA in plasmid pcDNA3.1 (#V790-20, Invitrogen, Thermo Scientific, USA) was firstly replaced by hRluc-neo fusion protein coding DNA fragment (RL) between Stu I and BstB I restriction sites to form vector pcDNA3.1-hRluc-neo. Following, the firefly luciferase (FL) coding DNA was cloned into pcDNA3.1-hRluc-neo between BamH I and EcoR I restriction sites to form vector pC3.1/Control report. Then, CTNNB1 5’-UTR coding DNA fragment was cloned into pC3.1/Control report between Hind III and Kpn I restriction sites to form vector pC3.1/5’-UTR report. CTNNB1 3’-UTR coding DNA fragment was cloned into pC3.1/Control report between Not I and Xba I restriction sites to form vector pC3.1/3’-UTR report. pC3.1/CDS report was generated by inserting FL coding DNA (without Stop Codon, FL-part) into pcDNA3.1-hRluc-neo between BamH I and EcoR I restriction sites, and next inserting CTNNB1 coding DNA (CTNNB1-part) between EcoR I and Xba I restriction sites. DNA fragment of hRluc-neo, Firefly luciferase and FL-part was amplified form plasmid pmirGLO Dual-Luciferase miRNA Target Expression Vector (#E1330, Promega, USA). CTNNB1 5’-UTR, CTNNB1 3’-UTR and CTNNB1-part coding DNA was amplified form cDNA of A549 cells. The oligonucleotide primers used in the PCR reaction for cloning of the indicated DNA are listed in **Supplementary Table S4**.

**Transfections and viral infection.** For plasmid transfection, cells were transfected with plasmids using Effectene Transfection Reagent (#301425, QIAGEN, Germany) according to the manufacture’s protocol. For siRNA transfection, cells were transfected by using HiPerFect Transfection Reagent (#301705, QIAGEN, Germany) according to the manufacture’s protocol. The sense sequence of nonsense control siRNA (NS control) and target gene siRNA are listed in **Supplementary Table S5**. For viral infection, cells were grown overnight to approximately 60% confluence and transiently infected with lentiviral particles at indicated MOI for 72 hours. Stable selection of CIRP overexpressed cells was supported by suppling culture medium with 2.0μg/mL puromycin (#P8833, Sigma-Aldrich, MO, USA).

**Cell proliferation assay.** Cell proliferation was assessed using a Cell Proliferation Kit I (MTT, #1146500700, Sigma-Aldrich, MO, USA). Briefly, indicated cells were plated at a density of 1×10^4^ cells/well in 6-well plate and incubated overnight. At each time point, MTT solution (1:10 dilution) was incubated with the cells for 3 hours, and then measured the absorbance at 570nm. For determine the IC_50_ of triptonide, Le-control and Le-CIRP A549 cells were plated at a density of 0.5×10^4^ cells/well in 6-well plate and incubated overnight. Then, the indicated concentration of triptonide were added and incubated for 48 hours for MTT assay. The IC_50_ value was deduced according the cell viability curves.

**Cell cycle analysis.** 5×10^5^ A549 and H460 cells were seeded in each well of a 6-well plate and transfected with indicated siRNA. 1×10^5^ CIRP stable overexpressed H1299 and H1650 as well as control cells were seeded in each well of a 6-well, respectively. After 72 hours culture, cells were harvested and fixed with cold 75% ethanol for 30 minutes. Then, cells were incubated with RNaseA with a concentration of 100mg/mL (R8021-25, Solarbio, Beijing, China) for 10 min and stained with propidium iodide 50 mg/mL (C0080, Solarbio, Beijing, China). Flow cytometry (FACSAriaTM II, BD Biosciences, Sparks, MD) was performed and the data was analyzed by Motif Software.

**Cell** **migration and** **invasion analysis.** The 24-well transwell plates (#3422, Corning, USA) with 8.0µm pore size were used to perform the migration analysis. Briefly, 48 hours after siRNA transfection, 1×10^4^ cells were seeded in the upper transwell chamber in 200µL serum-free medium. Then fueled the lower chamber with 600µL medium containing 10% FBS. For invasion analysis, the upper chambers were first precoated with Matrigel (#356234, BD, USA) and seeded with 4 × 10^4^ indicated cells as above described. After 24 hours incubation, carefully wiped out the cells which did not move through the pores with cotton wool. Cells on the undersurface of the upper chamber were fixed with 4% PFA and stained by 0.05% crystal violet. Then the number of cells for each chamber was counted in five random fields under an inverted microscope.

**Soft agar colony formation assay.** The colony formation ability of NSCLC cells was measured by soft agar colony formation assay. Briefly, we mixt equal volumes of 1.2% agarose solution and 2× cell culture medium (2× RPMI + 20% FBS), transferred 3mL of mixed solution to each well in 6-well plates allowing room temperature (RT) cool down to form the base agar layer. Then, we mixt equal volumes of 0.7% agarose solution and 2×cell culture medium, and followed 0.1mL of indicated cell suspension (5×10^4^/mL) was added to each 10mL of the mixed solution. 2mL of cell mixed solution was add to each well above the base agar layer and cooled down at RT. After 4 weeks incubation, the plates were stained with 0.5mL of 0.003% crystal violet for 1 hour. The colonies were counted by using a dissecting microscope.

**Gene expression microarray.** Gene expression profiles in A549 cells after transfection with siRNA 214/292 and NS-FAM control were compared using a GeneChip® PrimeView ™ Human Gene Expression Array (GCS 3000 7G; Affymetrix, USA). The sample preparation was performed by lysing three wells of independent transfection in 1mL of ice cold Trizol solution (#15596026, Thermo Scientific, MA, USA) as one pooled sample for microarray assay. The RNA extraction, quality control, Genechip hybridization as well as data acquisition were performed at the Beijing Cnkingbio Biotechnology Corporation (Beijing, China). Data files were analyzed by using SBC Analysis System software.

**RNA immunoprecipitation (RIP) assay.** For RIP assay, A549 cells were transiently infected with CIRP overexpression viral vector or respectively transfected with indicated luciferase reporters, and continually cultured for another 72 hours. Cells were washed twice with 10mL of ice-cold PBS and lysed with 1mL lysis buffer (150 mM NaCl, 1% IGEPAL CA-603, 0.5% DOC, 0.1% SDS, 50 mM Tris 100 U/ml Rnasin, 1 mm PMSF) for 10 min on ice [[3](#_ENREF_3)]. After centrifuge, 900μL supernatants were incubated with 100μL Pierce™ Protein G Agarose (#20398, Thermo Scientific, MA, USA) with rotating for 1 hour. Then, we collected and halved the supernatants (about 400μL) into two EP tubes. Following, 100μL pre-coated (Incubating with 20μL anti-CIRP or Normal rabbit anti-IgG for 6 h at 4 ℃) Protein G Sepharose beads were added and rotated for overnight at 4 ℃. After centrifuge, the beads were washed five times (0.5 mL wash, 5 min each) with Wash buffer (50 mM Tris-HCl, 150 mM NaCl, 1 mM MgCl2, 0.05% NP-40 and 100U/mL Rnasin)[[3](#_ENREF_3)]. Next, 200μL of proteinase K Buffer (0.5 mg/mL proteinase K and 0.1% SDS) was added and incubated at 55 °C for 30 min. Total RNA was isolated by using the RNeasy Mini Kit (#74104, QIAGEN, Germany) according to the manufacturer's protocol and resuspended in DEPC-treated water. The mRNA level of CTNNB1 and firefly luciferase was analyzed by qRT-PCR and normalized with input.

**Biotin pull-down assay.** Briefly, 50μg of cytoplasmic extraction were incubated with 50 pmol of biotin-label mRNA transcripts (Synthesized by GenePharma, Shanghai, China) for 1 hour at room temperature. The trolling protein complexes were then isolated with 50µL of Streptavidin conjugated solid matrix (DynabeadsM-280 Streptavidin, # 11205D, Invitrogen^TM^, Thermo Scientific, MA, USA). CIRP protein in the pull-down pellets was verified by Immunoblotting.

**Immuno-purification assay.** A549 cells were washed twice with Tap I buffer and lysed in 1mL of ice-cold Tap II buffer supplemented with Protease Inhibitor Cocktail (P8340, Sigma-Aldrich, MO, USA), and either 50 U/mL RNasin (N2111S, Promega, USA) or 10 mg/mL RNaseA (R8021-25, Solarbio, Beijing, China). Tap I and Tap II buffer were detailed in DOREEN’s report[[4](#_ENREF_4)]. Then, the Lysates were centrifuged at 13,000g for 10 min at 4°C to collect the supernatants. After pretreatment with Protein G Agarose for 1hour, 100μL pre-coated (Incubating with 20μL anti-CIRP or Normal rabbit anti-IgG for 6 h at 4 ℃) Protein G Sepharose beads were added and rotated for overnight at 4 ℃. After washed five times with Wash buffer, 100μL of ice-cold RIPA buffer was added for each bead pellet to eluate the protein. Immunoblotting was performed to verify the pulled-down proteins.

**mRNA decay analyses.** For CTNNB1 mRNA decay, A549 cells were firstly transfected with indicated siRNAs. For firefly luciferase mRNA decay, A549 cells were co-transfected with indicated siRNAs and luciferase reporters. 48 hours after transfection cells were treated with 5 mM Actinomycin D (#A4448, APEXBIO, USA) and harvested at indicated time points. Then, Total RNA was isolated and qRT-PCR was performed to analyze the relative mRNA levels.

**Luciferase reporter assay.** A549 cells were co-transfected with indicated siRNAs and luciferase reporters for 48 hours in 24-well plates. 100µL lysis buffer was added to each indicated well to collect the protein. Then 20µL of each supernatant were mixed with 100µL of Luciferase Assay Reagent II to evaluate the firefly luciferase activity, and the Stop & Glo® reagent was follow added to determine the Renilla luciferase activity (PR-E1910, Dual-Luciferase™ Reporter Assay Systems, Promega, USA). The Luciferase activities were measured by using Microplate Instrumentation (Cytation 5, BioTek, USA).

**Supplementary Table S1**. Relations between CIRP or CTNNB1 expression and clinicopathological parameters of the NSCLC patients.

| Variables | Total | CIRP | | *P* value | CTNNB1 | | *P* value |
| --- | --- | --- | --- | --- | --- | --- | --- |
|  |  | H | L |  | H | L |  |
| Gender  Male  Female  Age (years)  ≥60  <60  Death  No  Yes  Tumor grading  1  2  3  Tumor stage  I  II  III  T stage  T1  T2  T3 | 46  40  53  33  22  64  12  67  07  28  28  30  16  48  22 | 28 (60.9%)  20 (50.0%)  26 (49.0%)  22 (66.7%)  06 (27.3%)  42 (65.6%)  04 (33.3%)  40 (59.7%)  04 (57.1%)  13 (46.4%)  17 (60.7%)  18 (60.0%)  05 (31.3%)  27 (56.3%)  16 (72.7%) | 18 (39.1%)  20 (50.0%)  27 (51.0%)  11 (33.3%)  16 (72.7%)  22 (34.4%)  08 (66.7%)  27 (40.3%)  03 (42.9%)  15 (53.6%)  11 (39.3%)  12 (40.0%)  11 (68.7%)  21 (43.7%)  06 (27.3%) | 0.311  0.110  0.002*  0.238  0.476  0.039* | 20 (43.5%)  19 (47.5%)  24 (45.3%)  15 (45.5%)  05 (22.7%)  34 (53.1%)  06 (50.0%)  28 (41.8%)  05 (71.4%)  08 (28.6%)  18 (64.3%)  13 (43.3%)  06 (37.5%)  20 (41.7%)  13 (59.1%) | 26 (56.5%)  21 (52.5%)    29 (54.7%)  18 (54.5%)  17 (77.3%)  30 (46.9%)  06 (50.0%)  39 (58.2%)  02 (28.6%)  20 (71.4%)  10 (35.7%)  17 (56.7%)  10 (62.5%)  28 (58.3%)  09 (40.9%) | 0.709  0.988  0.024*  0.306  0.026*  0.311 |
| Lymph node metastasis | |  |  |  |  |  |  |
| No  Yes | 38  48 | 16 (42.1%)  32 (66.7%) | 22 (57.8%)  16 (33.3%) | 0.023* | 11 (28.9%)  28 (58.3%) | 27 (71.1%)  20 (41.7%) | 0.007* |

*, P < 0.05 statistically significant

**Supplementary Table S2.** List of antibodies.

| **Antigens** | **Source** | **Dilution** | **Supplier** |
| --- | --- | --- | --- |
| CIRP | Rabbit, mAb | 1:1,000 | #68522S, Cell Signaling Technology, USA |
| GAPDH | Rabbit, mAb | 1:2,500 | #5174S, Cell Signaling Technology, USA |
| CTNNB1 | Rabbit, mAb | 1:1,000 | #19807S, Cell Signaling Technology, USA |
| C-myc | Rabbit, mAb | 1:1,000 | #18583S, Cell Signaling Technology, USA |
| COX-2 | Rabbit, mAb | 1:1,000 | #12282S, Cell Signaling Technology, USA |
| CCND1 | Rabbit, mAb | 1:1,000 | #55506S, Cell Signaling Technology, USA |
| MMP7 | Rabbit, mAb | 1:1,000 | #3801S, Cell Signaling Technology, USA |
| VEGFA | Rabbit, pAb | 1:1,000 | #ab51745,  Abcam, USA |
| CD44 | Rabbit, mAb | 1:1,000 | #37259S, Cell Signaling Technology, USA |
| LARP3 | Rabbit, mAb | 1:1,000 | #5034S, Cell Signaling Technology, USA |
| PTBP1 | Rabbit, mAb | 1:1,000 | #57246S, Cell Signaling Technology, USA |
| YB-1 | Rabbit, mAb | 1:1,000 | #9744S, Cell Signaling Technology, USA |
| eIF4E | Rabbit, mAb | 1:1,000 | #2067S, Cell Signaling Technology, USA |
| eIF2α | Rabbit, mAb | 1:1,000 | #5324S, Cell Signaling Technology, USA |
| Anti-Rabbit-IgG (H+L)-HRP | Goat | 1:5,000 | #LK2001, Sungene Biotech, China |

**Supplementary Table S3.** The oligonucleotide primers used for qRT-PCR.

| Gene | Direction | Sequences (5′ to 3′) |
| --- | --- | --- |
| GAPDH  CIRP  WNT1  WNT2  WNT3A  CTNNB1  DDK3  LRP4  LRP5  LRP6  APC2  PP2A  GSK-3  AXIN  COX-2  CCND1  MMP7  CD44  AXIN2  TCF7  VEGFA  Firefly luciferase | Forward  Reverse  Forward  Reverse  Forward  Reverse  Forward  Reverse  Forward  Reverse  Forward  Reverse  Forward  Reverse  Forward  Reverse  Forward  Reverse  Forward  Reverse  Forward  Reverse  Forward  Reverse  Forward  Reverse  Forward  Reverse  Forward  Reverse  Forward  Reverse  Forward  Reverse  Forward  Reverse  Forward  Reverse  Forward  Reverse  Forward  Reverse  Forward  Reverse | ATTCAACGGCACAGTCAAGG  GCAGAAGGGGCGGAGATGA  AGGGCTGAGTTTTGACACCAA  ACAAACCCAAATCCCCGAGAT  CATCTTCGCAATCACCTCCG  GTGGCATTTGCACTCTTGG  GGCACAGGTTTCACTGTGGCTAAC  CCTGCCTCTCGGTCCCTGATAC  ATCGAGTTTGGTGGGATGGT  CGCTGTCGTACTTGTCCTTG  GTGCTGAAGGTGCTATCTGTCTGC  TGAACAAGACGTTGACTTGGATCTG  CACCCTCAATGAGATGTTCC  TGGTCTCATTGTGATAGCTG  GAGTGAAAAGAGCCCAGTGC  GGGGATTGGTTCAATCTTCA  GTTCCGGTCTGACGCAGTACA  GTCCATCAGGAAGTCCAGGT  CTTGTCAGCAGAGGAGAACTATG  CGTTGGAGGCAGTCAGAGG  CAGCTCAGCCAGGTCTTACACAG  CGTCCTGCAGGAACACCTTG  GTTCACCAAGGAGCTGGACCA  CATGCACATCTCCACAGACAGTAAC  CTGTTCCGAAGTTTAGCCTATAT  ACAAGAGGTTCTGCGGTTTA  TCACCCTGGGCCAGTTCAA  CAGTCAAACTCGTCGCTCACTTTC  CCGGGTACAATCGCACTTAT  GGCGCTCAGCCATACAG  TCAAGTGTGACCCGGACTGCCT  GCACGTCGGTGGGTGTGCAA  GCAACAGCGCATTTGAGCGGA  AATCAGCGTGCGACAGTTGCT  GAATTCTGCGCCCTCGGTT  CTGCCTCAGTCCGGGAGATA  ATTCGGCCACTGTTCAGACG  GACAACCAACTCACTGGCCTG  CTCGAGCCTACCCCCTGAAAG  GTTTAAACCAGGCTTTGAAAA  GCCTTGCCTTGCTGCTCTAC  TGATTCTGCCCTCCTCCTTCTG  CTGGTGCCCACACTATTTAGCTTC  CACCTACCTCCTTGCTGAGC |

| Gene | Direction | Sequences (5′ to 3′) | Restriction site | |
| --- | --- | --- | --- | --- |
| CTNNB1/cDNA  hRluc-neo  Firefly luciferase  CTNNB1 5’-UTR  CTNNB1 3’-UTR  FL-part  CTNNB1-part | Forward  Reverse  Forward  Reverse  Forward  Reverse  Forward  Reverse  Forward  Reverse  Forward  Reverse  Forward  Reverse | CGCGGATCCGCCACCatggctactcaagctg atttgatgga  ACGCGTCGACaaaggatgatttacaggtcagtatc  AAAAGGCCTatggcttccaaggtgtacgacc  CCGttcgaagtcccgctcagaagaactcgtcaaga  CGCGGATCCatggaagatgccaaaaacattaagaag  CCGGAATTCagaattacacggcgatcttgccgccct  CCCAAGCTTaagcctctcg gtctgtggca gcag  CGGGGTACCtgtccacgctggattttcaaaaca  AAGGAAAAAAGCGGCCGCatcatcctttagctgtattgtct  CTAGTCTAGAaatgaattaaaagtttaattctgaacc  CGCGGATCCatggaagatgccaaaaacattaagaa  CCGGAATTCcacggcgatcttgccgcccttcttgg  CCGGAATTCatggctactcaagctgatttgatgga  CTAGTCTAGAacagctaaaggatgatttacaggtcagta | | BamH I  Sal I  Stu I  BstB I  BamH I  EcoR I  Hind III  Kpn I  Not I  Xba I  BamH I  EcoR I  EcoR I  Xba I |

**Supplementary Table S4.** The oligonucleotide primers used for plasmids construction.

**Supplementary Table S5.** The oligonucleotides used for knockdown of indicated genes.

| Gene | Direction | Sequences (5′ to 3′) |
| --- | --- | --- |
| CIRP siRNA214  CIRP siRNA292  CIRP siRNA558  LARP3 siRNA  PTBP1 siRNA  YB-1 siRNA  CTNNB1 siRNA  NS control | Sense  Sense  Sense  Sense  Sense  Sense  Sense  Sense | GAGCAGGUCUUCUCAAAGUTT  GAUUUGGGUUUGUCACCUUTT  ggcUccagagacUacUaUaTT  GAAACAGACCUGCUAAUACTT^[^[^5^](#_ENREF_5)^]^  UCUUGCUGUCAUUUCCGUUUGCUGC^[^[^6^](#_ENREF_6)^]^  GGUCCUCCACGCAAUUACCAGCAAA^[^[^7^](#_ENREF_7)^]^  GGAUGUUCACAACCGAAUUTT  CCACACGAGUCUUACCAAGUUGCUU |

**Supplementary Figure Legends**

**Supplementary Fig. 1. The effectiveness of CIRP silencing and overexpression.** (A) Immunoblotting analysis detected CIRP expression in A549 cells transfected with CIRP siRNAs or NS control. A549 cells only treated with transfection reagent were used as mock controls. (B) Immunoblotting analysis detected CIRP expression in A549 and H460 cells transfected with combined siRNA214 and siRNA292. (C) CIRP was overexpressed and stable H1299 and H1650 cells were selected; the expression level of CIRP was determined by immunoblotting.

**Supplementary Fig. 2. Cell cycle analysis.** (A) Representative flow plots for cell cycle analysis of A549 and H460 cells after transfection with CIRP siRNA or NS control siRNA are shown. (B) Representative flow plots for cell cycle analysis of H1299 and H1650 cells stably overexpressing CIRP are shown.

**Supplementary Fig. 3. CIRP regulates the migration and invasion capability of A549 and H460 cells.** (A) Immunoblotting analysis detected the expression of CIRP in A549 and H460 cells stable infected with lentiviral vectors. Parental A549 and H460 cells served as controls. (B) Representative microphotographs showing the migration of CIRP overexpressed A549 and H460 cells. The migrated cell numbers are shown in histograms. (C) Representative microphotographs showing the invasion of CIRP overexpressed A549 and H460 cells. The invaded cell numbers are shown in histograms. Immunoblotting analysis of CIRP expression in A549 (D) and H460 (E) cells infected with Le-scrambled or Le-shCIRP at MOIs of 1and 10. All data are shown as the means ± SD from three independent experiments (**, P<0.01, *, P<0.05).

**Supplementary Fig. 4.** **Downregulation of CIRP Expression Abolishes the in vivo Tumorigenic Capacity of H460 Cells.** (A) H460 cells were subcutaneously inoculated into BALB/c nude mice after infection with Le-scrambled or Le-shCTNNB1 at an MOI of 10 (n = 10 for each group). Twenty-eight days after implantation, xenografts were harvested. (B) The weight of xenograft tumors was measured and shown in a scatter plot. (C) Immunohistochemical analysis of CIRP expression was performed on these xenografts. Representative images are shown (magnification ×40).

**Supplementary Fig. 5. CIRP Regulates the indicated Wnt/β-catenin Signaling Target genes in NSCLC Cells.** (A) Immunoblotting analysis detecting the indicated genes expression in A549 and H460 cells stable infected with CIRP overexpression lentiviral vectors. A549 and H460 cells stable infected with empty lentiviral vector were used as controls. (B) Protein levels of the indicated genes in CTNNB1-overexpressing and control A549 cells cotransfected with control or CIRP siRNA for 48 h. (C) MTT assay detecting the cell viability of Le-control and Le-CIRP A549 cells treated with different concentration of triptonide for 48 h.

**Supplementary Fig. 6. CTNNB1 Overexpression Correlates with the Poor Prognosis of NSCLC Patients.** (A) Representative microphotographs showing strong, moderate and weak staining of CTNNB1 in NSCLC tissues and their corresponding noncancerous lung tissues by IHC in tissue array (magnification × 40). (B, C) The percentages of strong, moderate and weak CTNNB1 expression in NSCLC samples (B) and corresponding noncancerous lung tissues (C) are shown in pie charts. (D) Kaplan-Meier analysis of the overall survival of NSCLC patients according to the expression level of CTNNB1 protein in NSCLC tissues (CTNNB1 high -expression, n = 39 and CTNNB1 low -expression, n = 47).

**Supplementary Fig. 7.** **Downregulation of CTNNB1 Expression Abolishes the in vivo Tumorigenic Capacity of A549 Cells.** (A) A549 cells were subcutaneously inoculated into BALB/c nude mice after infection with Le-scrambled or Le-shCTNNB1 at an MOI of 10 (n = 9 for each group). Twenty-eight days after implantation, xenografts were harvested. (B) The weight of xenograft tumors is measured and shown in a scatter plot. (C) Immunohistochemical analysis of CTNNB1 expression was performed on these xenografts. Representative images are shown (magnification ×40).

**Supplementary Fig. 8.** **CIRP Post-transcriptionally Regulates the Expression of CTNNB1.** (A) PCR analysis was performed to detect CTNNB1 mRNA, which was pulled down in RNA immunoprecipitation. cDNA of the A549 cell group (input) served as a positive control, and the IgG group served as a negative control. (B) The schematic diagram of the pC3.1/CDS reporter. (C) mRNA levels of firefly luciferase in the RIP assay were quantified by qRT-PCR. Data were normalized to the input group and presented as the means ± SD from three independent experiments. (D) Degradation of the firefly luciferase mRNA was analyzed by qRT-PCR in A549 cells cotransfected with pC3.1/CDS reporter and CIRP or control siRNAs. Data are shown as the means ± SD from three independent experiments. (E) Luciferase expression was measured in A549 cells cotransfected with CIRP or control siRNAs with the pC3.1/CDS reporter. Data are the means ± SD from three independent experiments.

**References**

1. Liao Y, Feng J, Zhang Y, Tang L, Wu S: **The mechanism of CIRP in inhibition of keratinocytes growth arrest and apoptosis following low dose UVB radiation.** *Molecular Carcinogenesis* 2016.

2. Li GQ, Fang YX, Liu Y, Meng FR, Wu X, Zhang CW, Zhang Y, Liu D, Gao B: **MALAT1-Driven Inhibition of Wnt Signal Impedes Proliferation and Inflammation in Fibroblast-Like Synoviocytes Through CTNNB1 Promoter Methylation in Rheumatoid Arthritis.** *Hum Gene Ther* 2019, **30:**1008-1022.

3. Mengxin, Lu, Qiangqiang, Ge, Gang, Wang, Yongwen, Luo, Xiaolong, Wei: **CIRBP is a novel oncogene in human bladder cancer inducing expression of HIF-1α.** *Cell death & disease* 2018.

4. Weidensdorfer D, Stöhr N, Baude A, Lederer M, Köhn M, Schierhorn A, Buchmeier S, Wahle E, Hüttelmaier S: **Control of c-myc mRNA stability by IGF2BP1-associated cytoplasmic RNPs.** *RNA* 2009, **15:**104-115.

5. Sommer G, Rossa C, Chi AC, Neville BW, Heise T: **Implication of RNA-binding protein La in proliferation, migration and invasion of lymph node-metastasized hypopharyngeal SCC cells.** *Plos One* 2011, **6:**10.

6. Zhang H, Wang D, Li M, Plecitá-Hlavatá L, D'Alessandro A, Tauber J, Riddle S, Kumar S, Flockton A, McKeon BA, et al: **Metabolic and Proliferative State of Vascular Adventitial Fibroblasts in Pulmonary Hypertension Is Regulated Through a MicroRNA-124/PTBP1 (Polypyrimidine Tract Binding Protein 1)/Pyruvate Kinase Muscle Axis.** *Circulation* 2017, **136:**2468-2485.

7. Lasham A, Samuel W, Cao H, Patel R, Mehta R, Stern JL, Reid G, Woolley AG, Miller LD, Black MA, et al: **YB-1, the E2F pathway, and regulation of tumor cell growth.** *J Natl Cancer Inst* 2012, **104:**133-146.
