# Supplementary material for: CIRP promotes the progression of non-small cell lung cancer through activation of Wnt/β-catenin signaling via CTNNB1
Source: J Exp Clin Cancer Res. 2021 Aug 31;40:275. doi: 10.1186/s13046-021-02080-9 (PMC8406911; doi:10.1186/s13046-021-02080-9)
Supplement: Supplementary file 2 — Additional file 2. [file 13046_2021_2080_MOESM2_ESM.pptx]

## Slide 1
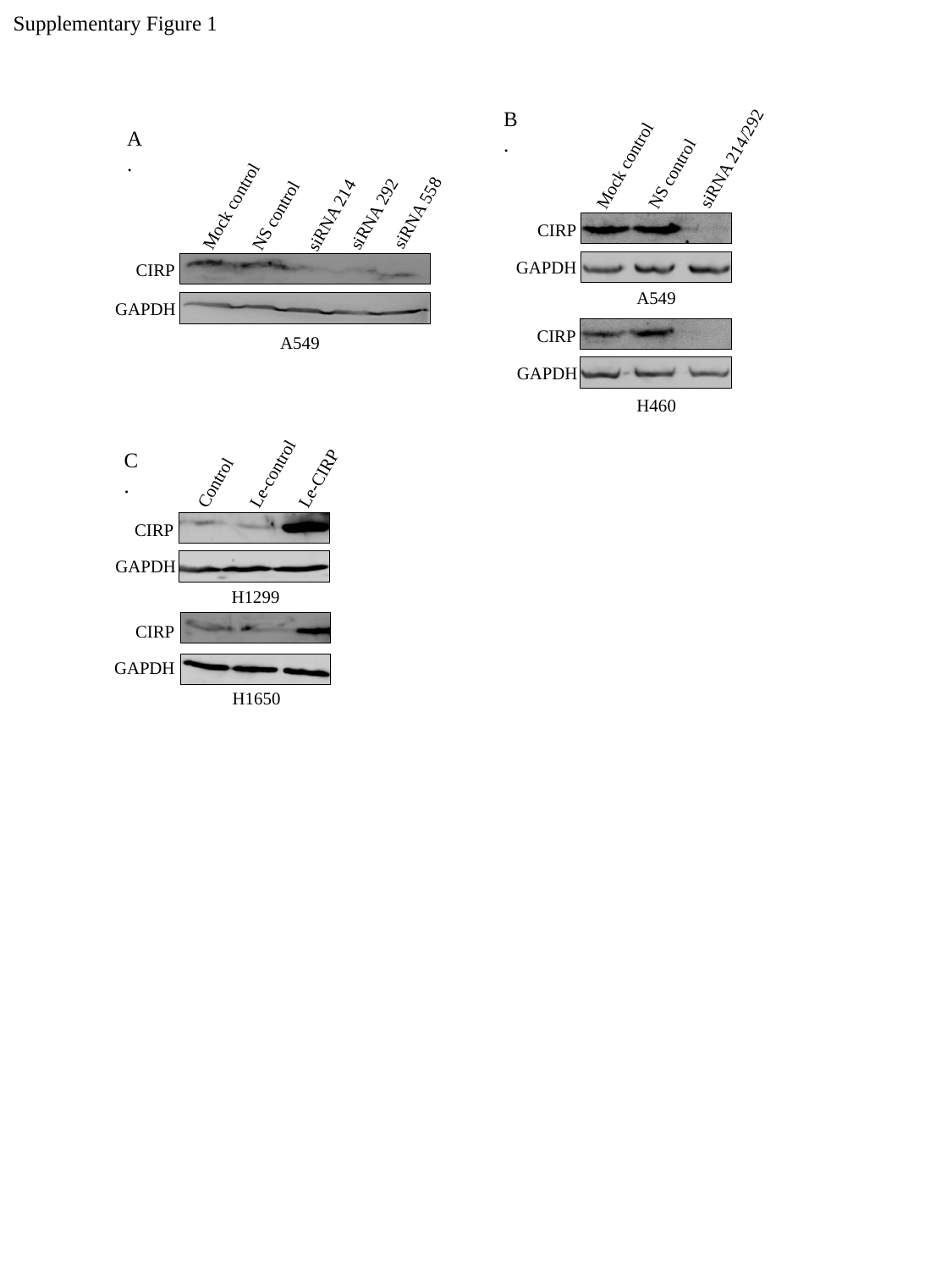

Supplementary Figure 1
NS control
siRNA 214/292
Mock control
CIRP
GAPDH
A549
CIRP
GAPDH
H460
B.
NS control
Mock control
siRNA 558
siRNA 292
siRNA 214
CIRP
 GAPDH
A549
A.
Le-control
Le-CIRP
Control
CIRP
 GAPDH
H1299
C.
CIRP
GAPDH
H1650

## Slide 2
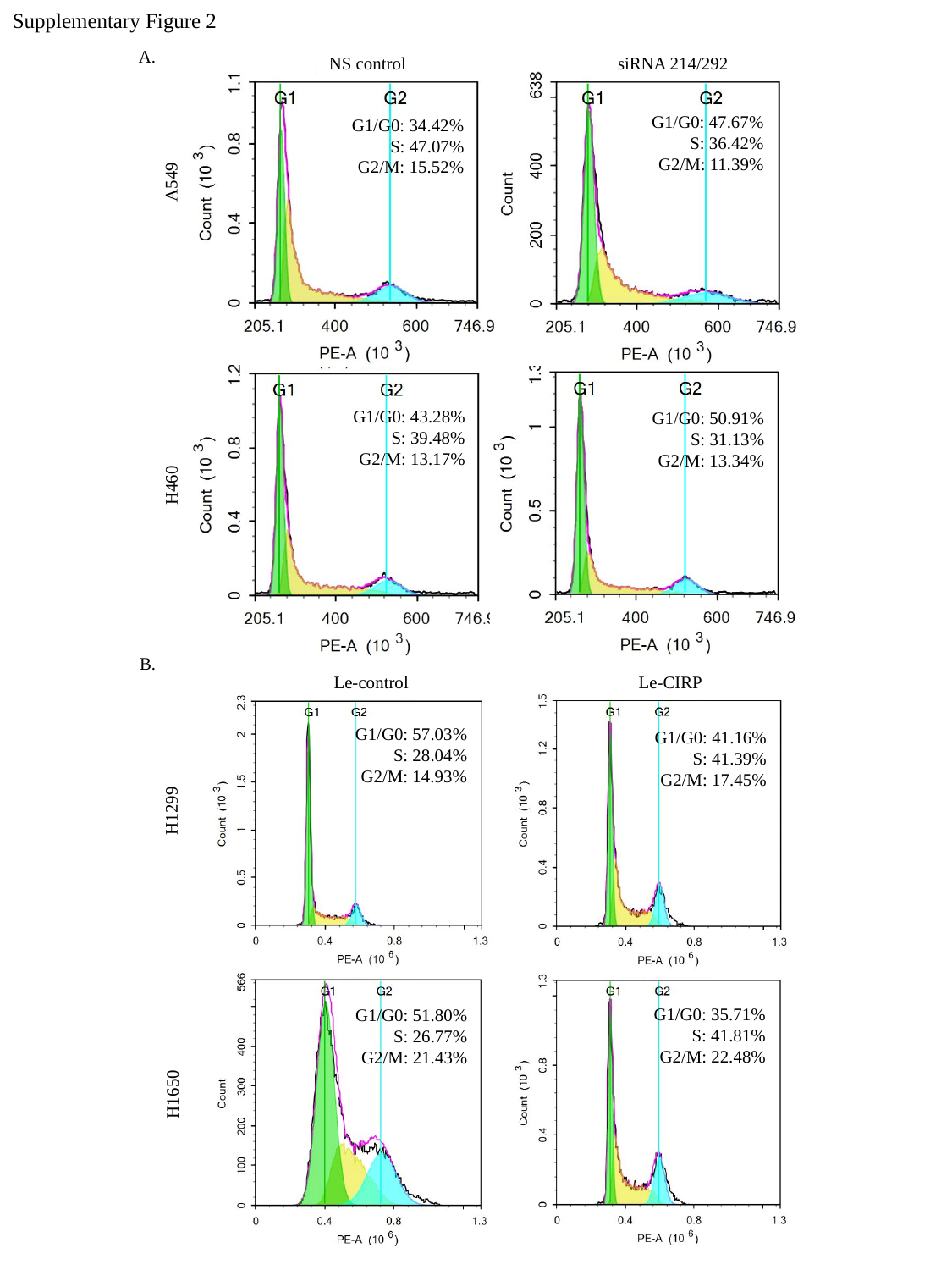

Supplementary Figure 2
A.
siRNA 214/292
NS control
G1/G0: 34.42%
 S: 47.07%
G2/M: 15.52%
G1/G0: 47.67%
 S: 36.42%
G2/M: 11.39%
A549
H460
G1/G0: 43.28%
 S: 39.48%
G2/M: 13.17%
G1/G0: 50.91%
 S: 31.13%
G2/M: 13.34%
B.
Le-control
Le-CIRP
G1/G0: 41.16%
 S: 41.39%
G2/M: 17.45%
G1/G0: 57.03%
 S: 28.04%
G2/M: 14.93%
H1299
G1/G0: 51.80%
 S: 26.77%
G2/M: 21.43%
G1/G0: 35.71%
 S: 41.81%
G2/M: 22.48%
H1650

## Slide 3
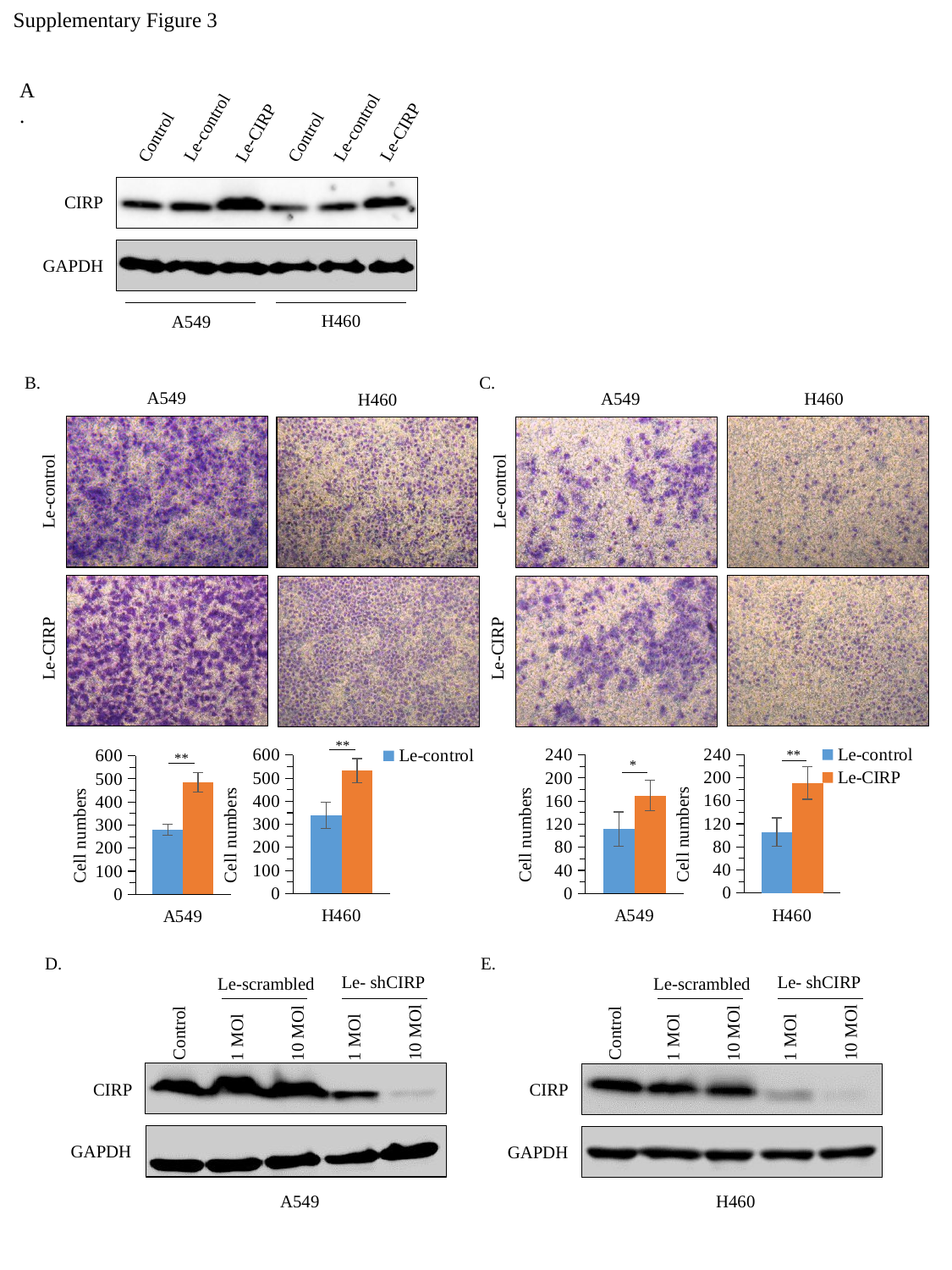

Supplementary Figure 3
A.
Le-control
Le-control
Le-CIRP
Le-CIRP
Control
Control
CIRP
 GAPDH
H460
A549
B.
A549
H460
Le-control
Le-CIRP
### Chart
| Category | Le-control
 | Le-CIRP |
|---|---|---|
| H460 | 338.8 | 533.2 |**
### Chart
| Category | Le-control | Le-CIRP |
|---|---|---|
| A549 | 279.0 | 484.2 |**
C.
H460
A549
Le-control
Le-CIRP
### Chart
| Category | Le-control | Le-CIRP |
|---|---|---|
| H460 | 105.66666666666667 | 191.0 |
### Chart
| Category | Le-control | Le-CIRP |
|---|---|---|
| A549 | 111.73333333333333 | 169.6 |*
**
D.
Le- shCIRP
Le-scrambled
10 MOl
Control
10 MOl
1 MOl
1 MOl
CIRP
GAPDH
A549
E.
Le- shCIRP
Le-scrambled
10 MOl
Control
10 MOl
1 MOl
1 MOl
CIRP
GAPDH
H460

## Slide 4
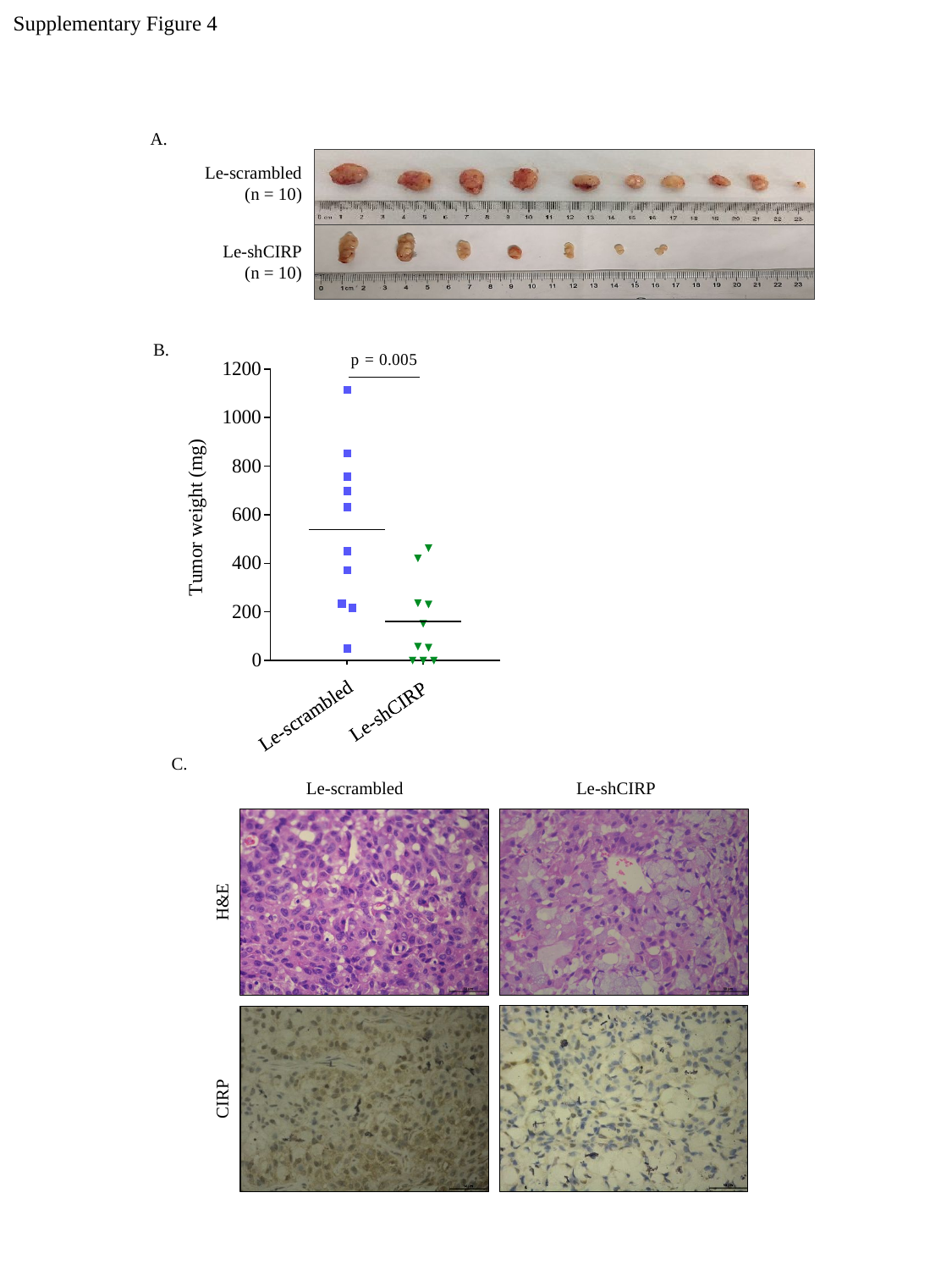

Supplementary Figure 4
A.
Le-scrambled
(n = 10)
Le-shCIRP
(n = 10)
B.
C.
Le-scrambled
Le-shCIRP
H&E
CIRP

## Slide 5
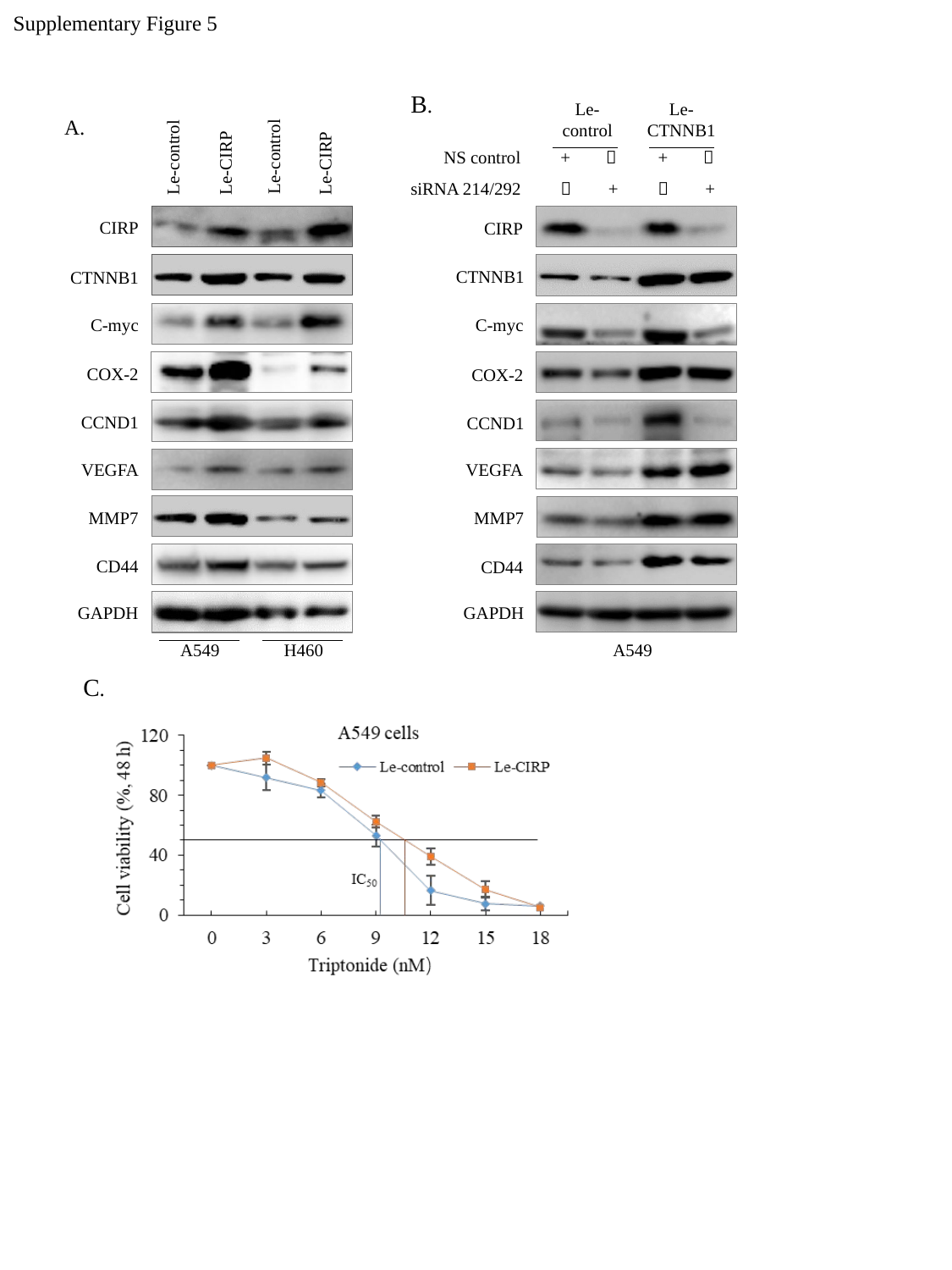

Supplementary Figure 5
B.
Le-
CTNNB1
Le-
control
NS control + － + －
siRNA 214/292 － + － +
CIRP
CTNNB1
C-myc
COX-2
CCND1
VEGFA
MMP7
CD44
GAPDH
A549
A.
Le-control
Le-control
Le-CIRP
Le-CIRP
CIRP
CTNNB1
C-myc
COX-2
CCND1
VEGFA
MMP7
CD44
GAPDH
A549
H460
C.

## Slide 6
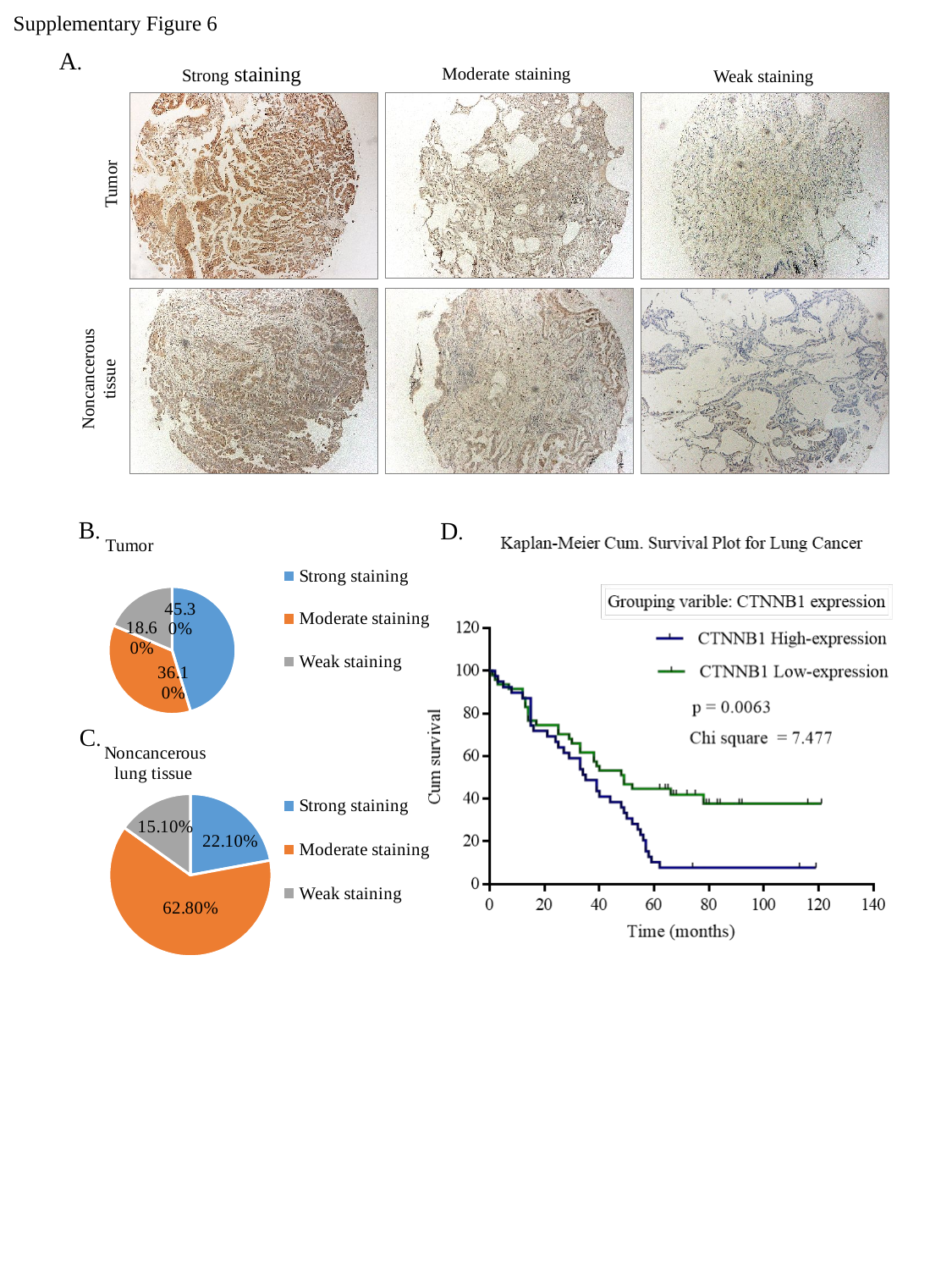

Supplementary Figure 6
A.
Moderate staining
Strong staining
Weak staining
Tumor
Noncancerous tissue
### Chart: Tumor
| Category | |
|---|---|
| Strong staining
 | 0.453 |
| Moderate staining
 | 0.361 |
| Weak staining | 0.186 |B.
C.
### Chart: Noncancerous
lung tissue
| Category | |
|---|---|
| Strong staining
 | 0.221 |
| Moderate staining
 | 0.628 |
| Weak staining | 0.151 |D.

## Slide 7
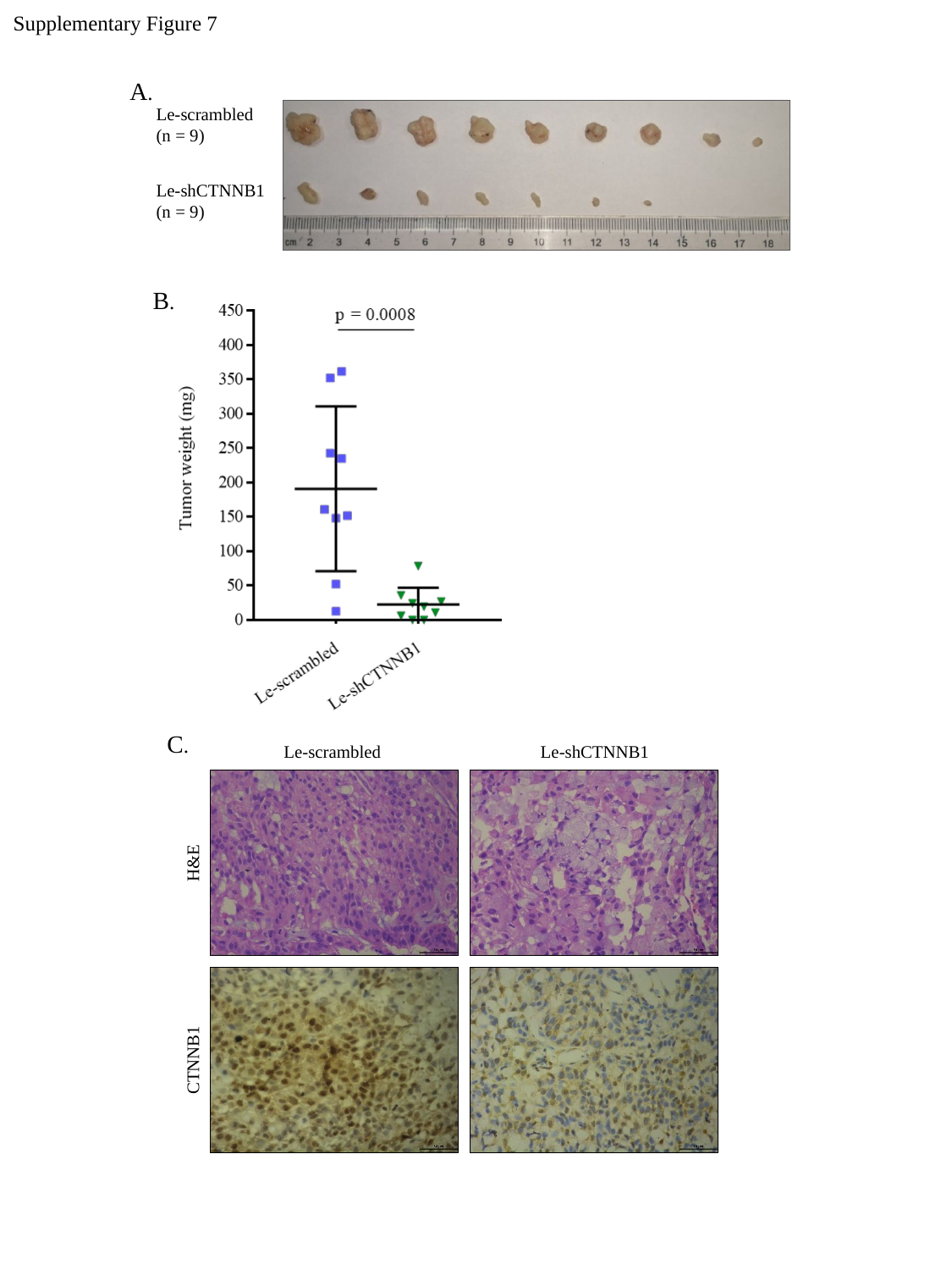

Supplementary Figure 7
A.
Le-scrambled
(n = 9)
Le-shCTNNB1
(n = 9)
B.
C.
Le-scrambled
Le-shCTNNB1
H&E
CTNNB1

## Slide 8
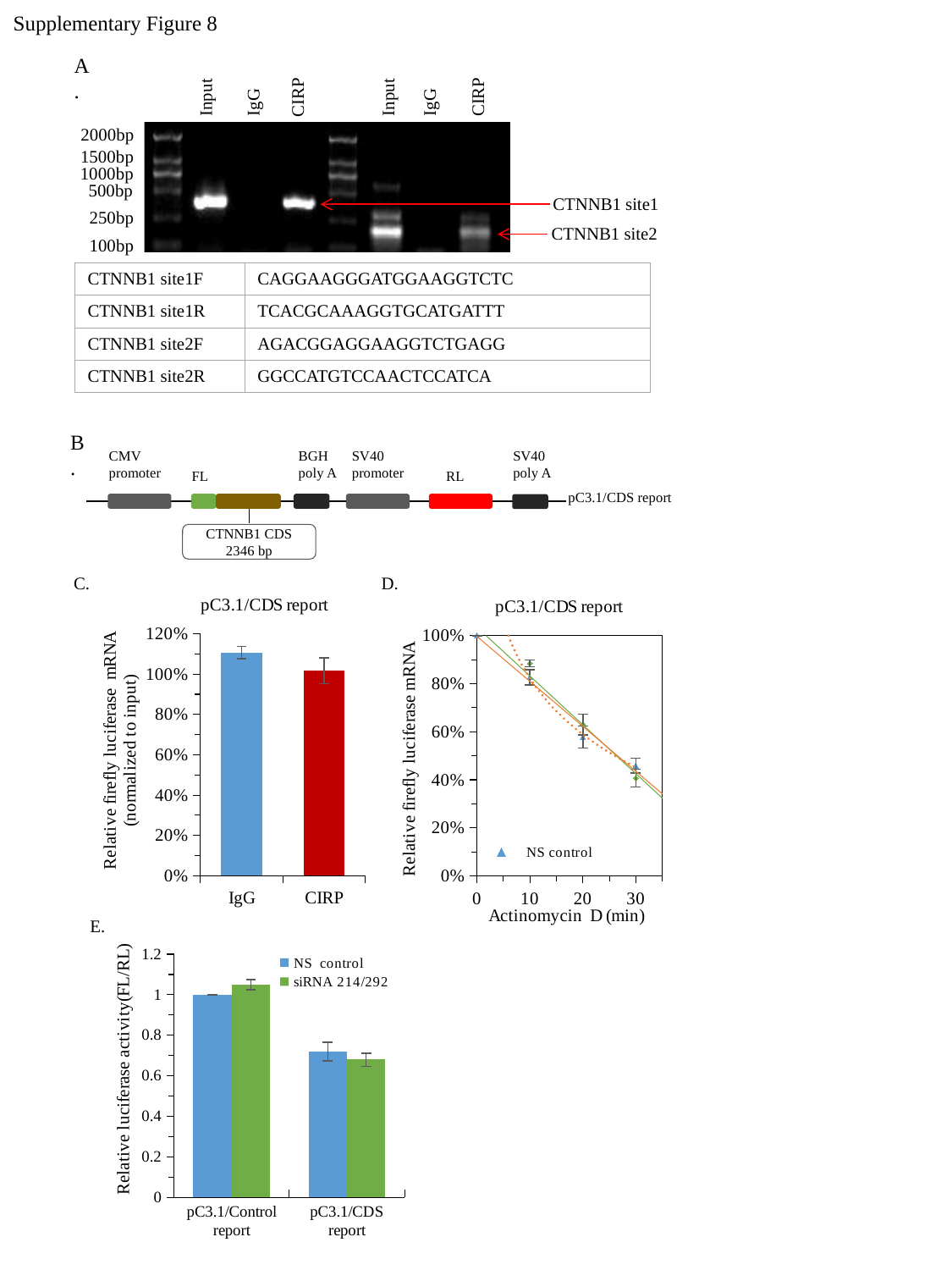

Supplementary Figure 8
A.
CIRP
CIRP
Input
Input
IgG
IgG
2000bp
1500bp
1000bp
500bp
250bp
100bp
CTNNB1 site1
CTNNB1 site2
| CTNNB1 site1F | CAGGAAGGGATGGAAGGTCTC |
| --- | --- |
| CTNNB1 site1R | TCACGCAAAGGTGCATGATTT |
| CTNNB1 site2F | AGACGGAGGAAGGTCTGAGG |
| CTNNB1 site2R | GGCCATGTCCAACTCCATCA |
B.
CMV
promoter
BGH
poly A
SV40
promoter
SV40
poly A
FL
RL
pC3.1/CDS report
CTNNB1 CDS
2346 bp
C.
### Chart: pC3.1/CDS report
| Category | IgG |
|---|---|
| IgG | 1.1057218102938802 |
| CIRP | 1.0167016929847998 |D.
### Chart: pC3.1/CDS report
| Category | NS control: pC3.1/Control report | NS control | siRNA214/292: pC3.1/Control report | siRNA214/292 |
|---|---|---|---|---|E.
### Chart
| Category | NS control | siRNA 214/292 |
|---|---|---|
| pC3.1/Control report | 1.0 | 1.05 |
| pC3.1/CDS report | 0.72 | 0.68 |
